# Small RNA Sequencing Reveals Differential miRNA Expression in the Early Development of Broccoli (*Brassica oleracea* var. *italica*) Pollen

**DOI:** 10.3389/fpls.2017.00404

**Published:** 2017-03-24

**Authors:** Hui Li, Yu Wang, Mei Wu, Lihong Li, Chuan Jin, Qingli Zhang, Chengbin Chen, Wenqin Song, Chunguo Wang

**Affiliations:** ^1^College of Life Sciences, Nankai UniversityTianjin, China; ^2^College of Horticulture and Landscape, Tianjin Agricultural UniversityTianjin, China

**Keywords:** broccoli (*Brassica oleracea* var. *italica*), high-throughput sequencing, miRNAs, microspores, microspore embryogenesis, male gametophyte

## Abstract

Pollen development is an important and complex biological process in the sexual reproduction of flowering plants. Although the cytological characteristics of pollen development are well defined, the regulation of its early stages remains largely unknown. In the present study, miRNAs were explored in the early development of broccoli (*Brassica oleracea* var. *italica*) pollen. A total of 333 known miRNAs that originated from 235 miRNA families were detected. Fifty-five novel miRNA candidates were identified. Sixty of the 333 known miRNAs and 49 of the 55 predicted novel miRNAs exhibited significantly differential expression profiling in the three distinct developmental stages of broccoli pollen. Among these differentially expressed miRNAs, miRNAs that would be involved in the developmental phase transition from uninucleate microspores to binucleate pollen grains or from binucleate to trinucleate pollen grains were identified. miRNAs that showed significantly enriched expression in a specific early stage of broccoli pollen development were also observed. In addition, 552 targets for 127 known miRNAs and 69 targets for 40 predicted novel miRNAs were bioinformatically identified. Functional annotation and GO (Gene Ontology) analysis indicated that the putative miRNA targets showed significant enrichment in GO terms that were related to plant organ formation and morphogenesis. Some of enriched GO terms were detected for the targets directly involved in plant male reproduction development. These findings provided new insights into the functions of miRNA-mediated regulatory networks in broccoli pollen development.

## Introduction

MicroRNAs (miRNAs) are a group of small endogenous non-coding RNAs, and widely distribute in animals and plants. In plants, mature miRNAs are 21–24 nt in length, and generated via a multi-step enzymatic process. Briefly, the primary miRNA transcripts (Pri-miRNAs) are transcribed by RNA polymerase II (Pol II). Pri-miRNAs are then processed to form imperfect fold-back structures, which are further cleaved by DICER-LIKE1 (DCL1) to produce stem-loop precursors (pre-miRNAs). Pre-miRNAs are further processed to produce miRNA/miRNA^*^ duplexes. Finally, mature miRNAs are released from these duplexes, and loaded into RNA induced silencing complex (RISC), to regulate their target genes by either transcript cleavage or translational inhibition (Bartel, 2004; Voinnet, 2009). Increasing evidence has demonstrated that miRNAs play crucial roles in almost all processes of plant growth, development and stress response. MiR172 regulates floral organ identity and flowering time by regulating APETELA 2 (AP2) transcription factors (Aukerman and Sakai, 2003; Chen, 2004; Wollmann et al., 2010). MiR156 as well as MiR172 control the vegetative phase change (Wu et al., 2009). MiR159 targeting MYB transcription factors is required for anther development, and acts as a molecular switch in seed development (Millar and Gubler, 2005; Alonso-Peral et al., 2012). MiR160, which targets auxin response factors (*ARFs*), functions in root development (Wang et al., 2005). MiR166 cleaves *ATHB15* mRNA, and functions in vascular development (Kim et al., 2005). MiR390, miR396, miR824, and miR319 are essential for leaf development (Schwab et al., 2006; Kutter et al., 2007; Liu et al., 2009). The members of at least nine miRNA families, including miR156, miR172, miR159 and other miRNAs, such as miR160, miR164, miR166/165, miR167, miR169, and miR319, perform crucial roles in flower development (Luo et al., 2013). However, the roles of many miRNAs, especially species-specific miRNAs, in plant development still need to be elucidated.

In flowering plants, development of male gametophytes is an important and complex biological process that is required to form pollen grains and is an indispensable part of sexual reproduction. The cytological characteristics of this process have been well defined. In brief, the sporogenous cells within the anther develop into pollen mother cells (PMCs) by mitosis. PMCs then undergo meiosis to form a tetrad of haploid microspores, which are held together by callose. The haploid microspores are released from tetrads after callose degradation. At this stage, only one nucleus exists in each microspore. Subsequently, individual microspore undergoes an asymmetric cell division to give rise to a vegetative cell and a generative cell, then develops into binucleate pollen grains. In flowering plants, such as cruciferous plants, the generative cell in turn further divides into twin sperm cells by mitosis. The microspores then develop into the trinucleate pollen grains. Finally, mature pollen grains are formed (Twell, 2011).

A series of regulators involved in male gametophyte development have been elucidated. *DYT1* and *AMS*, both encoding basic helix-loop-helix transcription factors, are crucial for tapetal differentiation and the formation of microspores (Seguí-Simarro and Nuez, 2008; Xu et al., 2010). *DUO1* is a germline-specific R2R3-MYB transcription factor that serves important functions in sperm cell specification by activating a germline-specific differentiation program (Borg et al., 2011). Five pollen-specific MIKC^*^ MADS box proteins function in later pollen development (Verelst et al., 2007). *PTC1* programs tapetal development and functions in pollen formation (Li et al., 2011). *ABCG26* and *ABCG15* are essential for pollen exine formation and male fertility (Quilichini et al., 2010; Zhao et al., 2015). Similarly, *LAP6/PKSA, LAP5/PKSB*, and *RPG1* are required for pollen exine formation. In addition, *LAP6/PKSA* and *LAP5/PKSB* can function at specific stages of microspore development (Guan et al., 2008; Kim et al., 2010). *PIRL1* and *PIRL9*, encoding members of a novel plant-specific family of leucine-rich repeat proteins, are essential for microspore differentiation (Forsthoefel et al., 2010). *BnMs3* participates in tapetum development, microspore release, and pollen-wall formation (Zhou et al., 2012). Several miRNAs such as miR158 and miR159 have been identified to function in pollen and/or anther development (Achard et al., 2004; Millar and Gubler, 2005; Luo et al., 2013; Ma et al., 2017). Because previous studies of the role of miRNAs in pollen and anthers focused on later stages of development, further investigations are required to understand how miRNAs impact early developmental stages of the male gametophyte.

Microspores are important intermediates in development of male gametophytes. In some Brassica plants (e.g., cauliflower, broccoli, Chinese cabbage and rapeseed) and Gramineous plants (e.g., maize, wheat, and barley), one notable feature of microspores is that they can deviate from their normal gametophytic development pathway and switch to embryogenesis *in vitro*, forming haploid embryos and homozygous double-haploid (DH) plants (Seguí-Simarro and Nuez, 2008). This process is referred to as microspore embryogenesis. It is a highly valuable tool to obtain homozygous plants in a significantly shorter period of time compared with the traditional breeding methods (Seguí-Simarro and Nuez, 2008). However, low microspore embryogenesis efficiency is one of the biggest bottlenecks. Emerging evidence has indicated that microspores at the uninucleate stage or early binucleate pollen grains are more easily induced into embryogenesis compared with those in other developmental stages (Keller et al., 1987; Ferrie, 2003), while the natural basis of this phenomenon is still largely unknown.

Broccoli is an important variant of *Brassica*, and one of the most important horticultural crops in *Brassicaceae*. Microspore embryogenesis is an important method to generate homozygous plants in broccoli. However, the regulation of microspore development is unknown. In the present study, uninucleate microspore, binucleate pollen grains, and trinucleate pollen grains, which represent three symbolic early developmental phases of male gametophyte in broccoli, were isolated. High-throughput small RNA sequencing and bioinformatics analysis were conducted to explore the miRNAs and their targets. MiRNAs possibly involved in the regulation of early pollen development of broccoli were identified. In addition, several miRNAs and their targets were further validated by experimental methods.

## Materials and methods

### Plant materials

Homozygous broccoli seeds named TNK-002 (stored in Tianjin Kernel Vegetable Research Institute, Tianjin, China) were planted in soil (Klasmann Deilmann GmbH, Germany) under controlled conditions with a 16/8 h light/dark cycle at 25° and 22°C, respectively. The 10-old day seedlings were transplanted in a greenhouse under growth condition as mentioned above. At approximately 70 days, the curds formed, which are mostly composed of flower buds with different sizes. The flower buds were isolated from the curds, and their sizes were measured by vernier caliper. The pollen in different developmental stages such as the uninucleate microspores, binucleate and trinucleate pollen grains, from differently sized flower buds were stained with DAPI fluorescent dye, and detected under a fluorescence microscope (Olympus BX53, Japan). Using the information obtained about the relationship between bud size and pollen developmental stage, three flower bud size ranges (3.0, 4.5, and 6.0 mm in length) were selected that would reproducibly yield uninucleate, binucleate pollen grains and trinucleate pollen grains, respectively.

### Isolation and purification of microspores, binucleate, and trinucleate pollen grains

Three groups of different-sized flower buds with uninucleate microspores, binucleate pollen grains and trinucleate pollen grains were collected. At least 50 flower buds per group were used. Then, stamens were isolated from the flower buds and deposited in 10-mL centrifuge tubes that contained 5 mL modified B5 culture medium. The stamens were then homogenized for 10 min to release single microspores, binucleate pollen grains or trinucleate pollen grains. In this process, the stamens were gently ground to avoid releasing single somatic cells. Subsequently, the mixtures that contained uninucleate microspores, binucleate pollen grains or trinucleate pollen grains were filtered with a 50-μm filter membrane to remove large impurities, and then filtered with a 40-μm filter membrane. The filtrate was transferred to other 10-mL centrifuge tubes and centrifuged at 1,000 g/min for 10 min. The remaining solid materials were resuspended with 5 mL modified B5 culture medium and centrifuged at 1,000 g/min for 10 min. Finally, the rinsed solid materials were collected, immediately frozen in liquid nitrogen, and stored at −80°C. To assess the purity of the isolated samples, approximately 50 μL of resuspended cells were stained with 5 μL DAPI. The stained cells were observed by fluorescence microscope (Olympus BX53, Japan) under UV light. In each sample, cells in at least 10 independent visual fields under the microscope were counted and analyzed based on their cytological characteristics.

### Construction and sequencing of small RNA libraries

Total RNAs were isolated using TRIzol reagent (Invitrogen, USA). RNA samples with high purity (OD_260_/_280_ = 1.8–2.2) and high integrity (RNA integrity number, RIN > 8.0) were used to construct small RNA libraries by TruSeq Small RNA Library Prep Kit (Illumina, USA) according to the manufacturer's protocols. In brief, total RNAs were subjected to 15% denaturing polyacrylamide gel electrophoresis. Small RNA fractions of 18–30 nt were isolated from the gel and purified. The 5′ and 3′ adapters were ligated to the isolated small RNAs by T_4_ RNA ligase (TaKaRa, Japan), and then converted to cDNAs which were further used as samples to conduct RT-PCR amplification. The adapter sequences and RT-PCR primers were showed in Table S1. The PCR products were purified and subjected to deep sequencing by HiSeq™ 2000 (Illumina, USA) [Beijing Genomics Institute (BGI), China]. Three repeats were conducted in the sequencing process. 49-nt raw reads were produced by the sequencing system.

### Classification and annotation of small RNA

Raw reads of small RNAs were obtained via Solexa sequencing. Low-quality reads and contaminants in the raw reads, for example, reads that were less than 18 nt in length, adaptor-only and polyA reads, were discarded. The adaptor sequence of each read was trimmed. Filtered high-quality reads that were 18–30 nt in length were designated as clean reads and further assembled into unique reads. The clean reads and unique reads were used in the following analysis. The length distributions of clean reads in uninucleate microspores, binucleate and trinucleate pollen grains were analyzed. Pairwise comparisons of common/specific clean and unique reads were also conducted. Then, all unique reads were aligned with structural non-coding RNAs (rRNA, tRNA, snRNA, snoRNA, etc.) deposited in the GenBank and Rfam databases. Reads that matched with the sequences in these two databases were excluded in the further analysis. Subsequently, the unique reads were subjected to Blastn analysis against plant miRNAs, particularly *Arabidopsis* and other Brassicaceae plant miRNAs deposited in miRBase 18.0 (http://www.mirbase.org/) to identify the conserved miRNAs. The unique reads detected in each small RNA library were annotated in accordance with the criteria: ribosomal RNA > known miRNA > repeat > extron > intron.

### Prediction of novel miRNAs

The unannotated reads and reads from the introns were aligned to broccoli EST data (Accession number: PRJNA361430) to identify potentially novel miRNAs. Sequences >100 bp surrounding the matched region were extracted, and utilized to predict pre-miRNA candidates using the Mireap program (https://sourceforge.net/projects/mireap/). Parameters were as follows: Minimal miRNA sequence length (18 nt); Maximal miRNA sequence length (25 nt); Minimal miRNA reference sequence length (20 nt); Maximal miRNA reference sequence length (23 nt); Maximal copy number of miRNAs on reference (20 nt); Maximal free energy allowed for a miRNA precursor (−18 kcal/mol); Maximal space between miRNA and miRNA^*^ (300 nt); Minimal base pairs of miRNA and miRNA^*^ (16 nt); Maximal bulge of miRNA and miRNA^*^ (4 nt); Maximal asymmetry of miRNA/miRNA^*^ duplex (4 nt); Flank sequence length of miRNA precursor (20 nt). The predicted pre-miRNA sequences were further assessed by M-fold (Zuker, 2003), and only structures with the lowest free energies were selected. In addition, to separate novel miRNA candidates from possible siRNAs, small RNA read distribution was analyzed using Blastall and Omega 2.0 softwares. Small RNAs with wide distribution on the precursor sequences and having reads that almost equally map to both plus and minus strands were excluded.

### miRNA expression profile and differential expression analysis

In small RNA deep sequencing, the count of clean reads originating from each miRNA represents the expression abundance or level of the corresponding miRNA. At least 16-nt overlap was required to confirm a read that generate from a certain miRNA. To explore the expression patterns of miRNAs in three different developmental phases of broccoli pollen, the frequency of each miRNA was normalized to the same order of magnitude according to the formula: Normalized expression = actual miRNA count/total count of clean reads × 1,000,000. The normalized read count in miRNAs from which no reads were detected, was set at 0.01. The differential expression level of each miRNA in two arbitrary developmental phases was evaluated by the fold change of the normalized expression level. The log base 2-fold change was calculated as follows: the log base 2-fold_change = log_2_ (A/ B) (A and B represent the normalized expression level of miRNA in any two developmental phases). Then, statistical analysis was performed according to Poisson distribution. The *P*-value was calculated and further corrected by Bonferroni Correction. For the identification of significantly expressed miRNAs, the criteria was used as if (log base 2-fold change ≥1 or ≤–1) and (corrected *P* < 0.05). A hierarchical cluster analysis of all known miRNAs was done by the package “gplots” of the R project according to the log base 2-fold_change of the normalized expression levels in two arbitrary developmental phases (http://www.r-project.org/).

### miRNAs target prediction

Conserved and novel miRNAs were aligned with the broccoli ESTs to predict the potential targets of these miRNAs. If one EST sequence satisfied the parameters that were suggested by Allen et al. (2005) and Schwab et al. (2005), the EST was considered as a putative target of a specific miRNA. In addition, the Gene Ontology (GO) analysis of putative targets was conducted. Target gene candidates from broccoli uninucleate microspores, binucleate pollen grains and trinucleate pollen grains were mapped to GO terms in the database (http://www.geneontology.org/), respectively. Then, the gene numbers per term were calculated. The hypergeometric test was conducted to identify the GO terms in target gene candidates that were significantly enriched compared with the reference gene background (broccoli ESTs). The calculating formula is:

(1) $$\text{P = 1}-\sum_{i\;=\;0}^{m-1}\frac{\left(\underset{i}{M}\right)\left(\underset{n-i}{N-M}\right)}{\left(\underset{n}{N}\right)}$$

In the formula, *N* is the number of all genes with GO annotation; *n* is the number of target gene candidates in *N*; *M* is the number of all genes that are annotated to a certain GO term; *m* is the number of target gene candidates in *M*. Then, the Bonferroni Correction is used for the *P*-value to obtain a corrected *P*-value. GO terms with corrected *P* < 0.05 are defined as significantly enriched in target gene candidates. To further describe the enrichment level of each GO term, the formula [Enrichment = (*n*/*N*)/(*m*/*M*)] is used.

### Validation of miRNAs and miRNA targets

Total RNA was isolated from microspores, binucleate and trinucleate pollen grains by TRIzol reagent (Invitrogen, USA) according to the manufacturer's instructions. RNAs free of contaminated genomic DNAs were reverse-transcribed using miRNA-specific stem-loop primers (Table S1). The subsequent PCR amplification and re-sequencing analysis of miRNAs were conducted as described by Geng et al. (2014).

The expression levels of miRNAs and miRNA targets were further validated by real-time quantitative RT-PCR (qRT-PCR). Specific primers were designed based on the sequences of miRNAs and corresponding targets (Table S1), respectively. Faststart Universal SYBR Green Master (Roche, Germany) was used in all qRT-PCR experiments. Small nuclear RNA U6 was used as an internal reference in miRNA expression level analysis, while the *actin* gene of broccoli was used as an internal control in target expression level analysis. The relative expression levels of miRNAs and targets were calculated by the comparative 2^−ΔΔCT^ method according to the manufacturer's recommendations. To ensure the reliability of quantitative analysis, three batches of independently isolated RNAs from the three different developmental phases of broccoli pollen were used, and three technological replicates were performed.

### Identification of miRNA target cleavage sites by 5′ RLM-RACE

To validate miRNA targets, a modified 5′ RLM-RACE (RNA ligase-mediated rapid amplification of cDNA ends) was conducted using a 5′-Full RACE Kit (TaKaRa, Japan) in accordance with the manufacturer's instructions with some modifications. Total RNA that was isolated from uninucleate microspores, binucleate and trinucleate pollen grains were ligated to the 5′ RNA adaptors by T_4_ RNA ligase (TaKaRa, Japan). A reverse transcription reaction was performed with 9-nt random primers. Subsequently, a nested PCR reaction was performed. In the first PCR reaction, the reverse transcription product was amplified using the 5′ RNA adaptor outer primers and gene-specific outer primers (Table S1). In the second PCR reaction, the first PCR products (1 μL) were amplified as templates with an inter primer combination (5′ RNA adaptor inter primers and gene-specific inter primers) (Table S1). After amplification, 5′ RACE products were gel-purified and cloned using the T-A cloning method by Mighty TA-cloning Kit (TaKaRa, Japan). A minimum of 10 independent clones from each PCR reaction were randomly selected and sequenced.

## Results

### Uninucleate microspores, binucleate, and trinucleate pollen grains isolation

**Figure 1:**
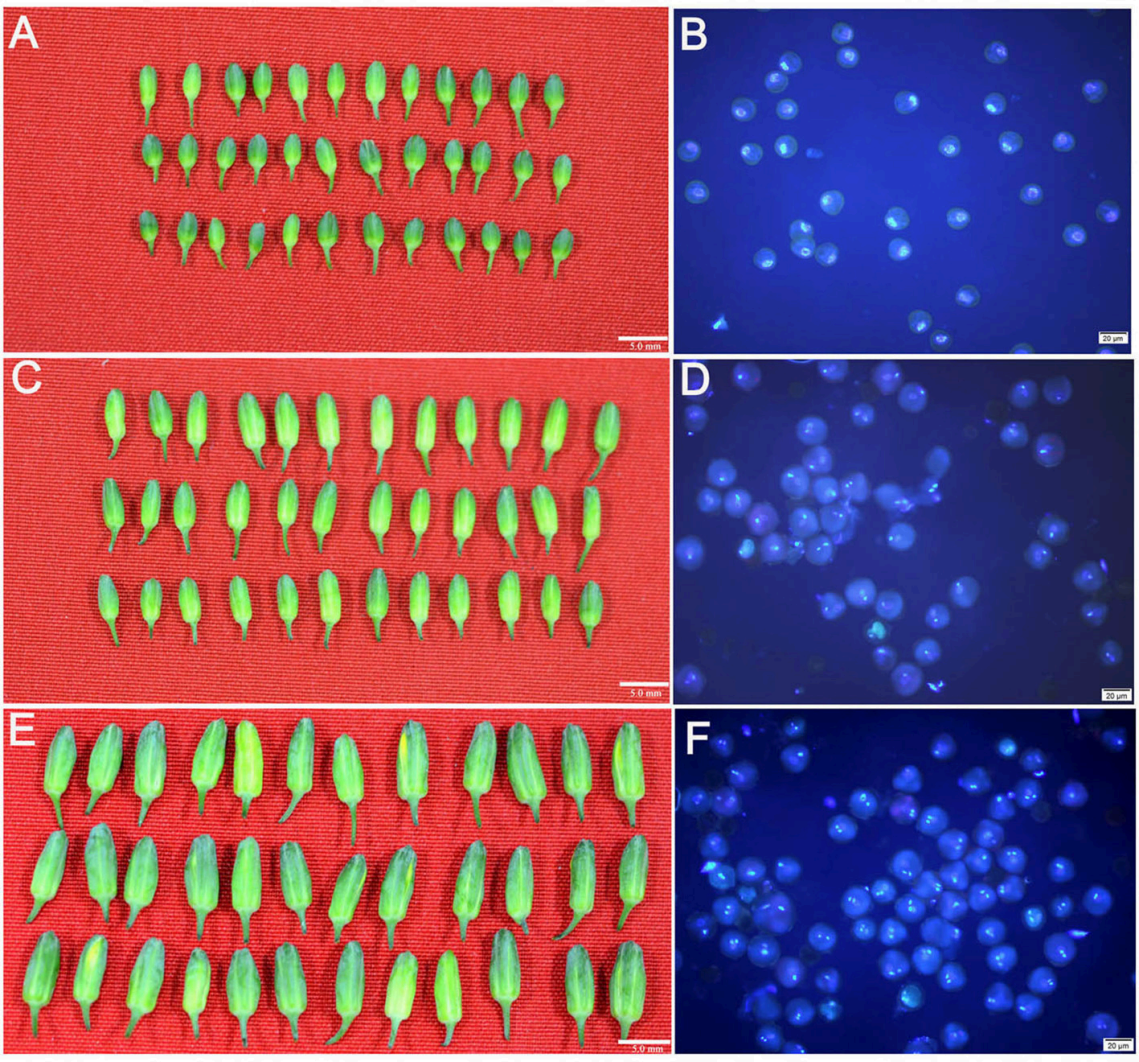
**Uninucleate microspores, binucleate pollen grains and trinucleate pollen grains isolated form the flower buds with different sizes in broccoli. (A,C,E)** indicated that the broccoli flower buds were average 3.0, 4.5, and 6.0 mm in length, respectively (Bar = 5.0 mm). **(B,D,F)** indicated the isolated uninucleate microspores, binucleate pollen grains and trinucleate pollen grains from the corresponding flower buds, respectively (Bar = 20 μm).

Microspores, binucleate pollen grains and trinucleate pollen grains, were isolated from broccoli flower buds (Figure 1). Statistical analysis indicated that the purity of the isolated uninucleate microspores and unmatured pollen grains in binucleate and trinucleate phases exceeded 95% (Table S2). This result indicated that the quality of the isolated samples was sufficient to conduct the following analysis.

### Overview of small RNA sequencing results

Three small RNA libraries from the corresponding isolated samples were constructed and sequenced by Solexa technology (Accession number: PRJNA361414). In total, 21,659,809, 20,439,333, and 21,429,804 raw reads generated from the RNA of uninucleate microspores, binucleate pollen grains and trinucleate pollen grains, respectively. After removing low-quality reads, contaminants and adaptors, 21,429,050 clean reads representing 7,160,154 unique sequences in uninucleate microspores, 20,173,912 clean reads representing 6,299,777 unique sequences in binucleate pollen grains and 21,209,081 clean reads representing 6,952,258 unique sequences in trinucleate pollen grains were obtained (Table S3). Pairwise comparisons of the small RNAs from the three small RNA libraries were conducted. Consisted with reported investigations, the vast majority of unique reads are non-overlapping. In uninucleate microspores and binucleate pollen grains, only 14.79% unique reads were shared in the two developmental phases. In uninucleate microspores and trinucleate pollen grains, 43.28 and 41.59% unique reads were specific in uninucleate and trinucleate phase, respectively. Similarly, the majority of the unique reads, which were detected in binucleate and trinucleate pollen grains, were specific to the binucleate (39.95%) pollen grains or trinucleate (45.59%) pollen grains. Only a small proportion of these reads (14.46%) was shared in these two developmental phases (Figure S1). All unique reads were then further annotated. Only a few reads were annotated as ribosomal RNAs, tRNAs, snRNAs, snoRNAs, miRNAs, and other previously known RNAs. More than 97% of the reads were unannotated. The length distributions of all of the clean reads were also explored. The majority of the reads were 18–24 nt in length. The highest abundance was found for reads that were 24 nt in length followed by 21 nt, 22 and 23 nt in length (Figure S2).

### Identification of conserved miRNAs

In total, 333 known miRNAs were detected in the three early stages of broccoli pollen development. These miRNAs originated from 235 different miRNA families. The majority of these conserved miRNAs (325/333) were simultaneously detected in three samples. However, the expression abundance of each miRNA varied (Tables S4, S5). The members in the miRNA families, such as miR167, miR166, miR156/157, miR397, miR165, miR158, miR168, miR2199, miR2911, miR5782, and miR6300 families, had a high expression abundance (clean read count of miRNA > 10,000) in all three developmental phases. MiRNAs, such as miR159, miR162, miR172, miR390, miR2916, and miR391, showed a moderate expression abundance (clean read count of miRNA > 1,000), whereas most miRNAs, especially, miR169i, miR169j, miR4221, miR399d, miR5026, miR5642a, miR5642b, miR775, and miR828 displayed a low expression abundance (clean read count of miRNA < 1,000) (Figure S3, Table S4, S5). Further analysis indicated that only one gene was detected in most miRNA families. At least two genes were detected in the other 25 miRNA families. The miR156/157, miR166, miR167, miR169, miR172, miR395, and miR399 families had more than four members, although high or moderate expression abundance of several these miRNA families was not detected (Figure S4).

### Prediction of novel miRNAs

A total of 55 novel miRNA candidates were observed. Among these novel miRNAs, 31, 28, and 26 predicted novel miRNAs were detected in the uninucleate microspores, binucleate pollen grains and trinucleate pollen grains, respectively. The lengths of these newly predicted miRNAs ranged from 20 to 23 nt, among which 21-nt miRNAs were dominant. The negative minimum folding free energy (MFE) of these miRNA precursors ranged from −20 kcal/mol to −85.3 kcal/mol. The average free energies were approximately −40.37 kcal/mol (Table 1). Each novel miRNA precursor can form regular stem-loop structures. The star sequences (miRNA^*^) of 28 novel miRNA candidates were observed, which further confirmed the existence of these miRNAs. Further analysis indicated that the expression patterns of most of the predicted novel miRNA varied. Forty-one of the 55 predicted novel miRNAs were specifically expressed in the uninucleate microspores, binucleate pollen grains or trinucleate pollen grains. Among them, 15 predicted novel miRNAs were only detected in the uninucleate microspores, 13 predicted novel miRNAs were specifically expressed in the binucleate pollen grains. Similarly, 13 predicted novel miRNAs were only detected in the trinucleate pollen grains (Table S6). Four predicted novel miRNAs (bol-miR01, bol-miR03, bol-miR24, and bol-miR25) were detected in the uninucleate microspores and binucleate pollen grains but not in the trinucleate pollen grains. Bol-miR27, bol-miR30, bol-miR33, and bol-miR38 were only detected in the binucleate and trinucleate pollen grains. However, no predicted novel miRNAs were specifically expressed only in uninucleate microspores and trinucleate pollen grains. Nevertheless, six predicted novel miRNAs (bol-miR02, bol-miR04, bol-miR7, bol-miR9, bol-miR18, and bol-miR22) were simultaneously detected in the three developmental phases (Table S6). In total, 49 of the 55 predicted novel miRNAs displayed specific expression patterns in the three different developmental phases of broccoli pollen.

**Table 1:** **The predicted novel miRNAs in broccoli uninucleate microspores, binucleate pollen grains, and trinucleate pollen grains**.

**Novel miRNAs**	**Sequence (5′-3′)**	**MFE (kcal/mol)**	**Novel miRNAs**	**Sequence (5′-3′)**	**MFE (kcal/mol)**
bol-miR01	GGAATGTTGTTTGGCTCGAAG	−20	bol-miR29	TAATCATGTTTAGACTTAGATCA	−46.7
bol-miR02	AGATATTAGTGCGGTTCAATC	−44.5	bol-miR30	GGACGTGCTGTAGGAGTAACCC	−28.7
bol-miR03	GGCCGTGGGATGTGGATGGCA	−45.5	bol-miR31	GGAGGATGACGGAGAAGGAGCA	−60.5
bol-miR04	ATGCACTGCCTCTTCCCTGGC	−38.2	bol-miR32	TCTGGTGTAGACGTGGTATC	−48.9
bol-miR05	GTTCTTAAAGTAGATCTTCGGAA	−44.7	bol-miR33	ACAGCTCTGCTCCATCTGTGA	−85.3
bol-miR06	AATGAAATAAGAAGGCGAGGATT	−37.3	bol-miR34	TGGCTGCTGGAATTGATGGTC	−34.7
bol-miR07	TGCATCAACTGAATCGGAGCC	−73.5	bol-miR35	TCCCTTTGGATGTCGTCTTGTG	−30.1
bol-miR08	TCTCAAACCGTGATGATGGACG	−30.1	bol-miR36	GTGTTGTTTGACTTGAGATGACG	−44.1
bol-miR09	TCTGAAACTTTGTGACATTGCAG	−57.4	bol-miR37	ACTTCACATTGTGGTCGTCTGA	−37.5
bol-miR10	TAGCTAGTAGATGTTGTCGTG	−22.8	bol-miR38	CCAGATCTGGGATTGGCCAAC	−43.9
bol-miR11	CTTCGTGTAAATGATTTTCCTT	−33.3	bol-miR39	TCTCAGTGGATTTCGAATGGA	−29.6
bol-miR12	CAGCGAAGAGGATGTAGCGGAG	−36.1	bol-miR40	CAGCTGTAGAGTGCTGGAAGGA	−25.4
bol-miR13	AGAACTCATGAGAAGGCTTGGTG	−29.6	bol-miR41	CGATTGTGGAAGAAAGTGGA	−34.2
bol-miR14	GGAAGACCGGTGAAACTCATCTC	−29.3	bol-miR42	GGAGAGTCGGCGTGGCATCAAG	−71
bol-miR15	ACCCTTCTCACGCAGATCAAC	−30.7	bol-miR43	AATCGGCTTGTAGCAGTGGCA	−55.2
bol-miR16	GATTGACGACGACGAGGGAGACG	−75.2	bol-miR44	CGACAGCTTCTCGTCGGTCGATA	−45
bol-miR17	CGGAGGAAACGGGTTCTCGGG	−25.8	bol-miR45	CTAAGCAGGATCCAAAGACGTT	−28.6
bol-miR18	TGGATATGATGAAATGGCATA	−29	bol-miR46	AGGTGGTTATTGGAGTCGGTTTA	−40.7
bol-miR19	AGGCAGCGAAGTAGGACTGGCTT	−38.4	bol-miR47	CAAGTTGTAGGTTAGTTTTGGCA	−34.2
bol-miR20	GAGGTATGGGACGATGGTGG	−49.7	bol-miR48	AGGAGACTGTTTATGTAAGGCTA	−28.1
bol-miR21	GCTTGTCTCTGAGATCCCGGCG	−28.5	bol-miR49	AGGACTATAGGCGAAGCGGGGTT	−37.8
bol-miR22	TTGTGCAAGACTAAGAAGCAA	−46.7	bol-miR50	CACCCTTCTCACGCAGATCAA	−35.7
bol-miR23	TGGCTAAATCCAGATATGTCG	−43.9	bol-miR51	TCCCTTTGGATGTCGTCTTGT	−30.1
bol-miR24	GCTTCTAGTGCGATCGGGTTCGG	−49.3	bol-miR52	CGTCATTTGGATCCATCGGG	−33.7
bol-miR25	GCCATGGCGGAAGAGTTTTTATC	−36.1	bol-miR53	GTGCTTATTGACGGTCTTGT	−32.5
bol-miR26	ACTTGTTTCGTCGGTATGTCG	−51.6	bol-miR54	AGGACTTGTCTTGGATAGGTATA	−29.9
bol-miR27	GACGGTTCTTAGCTTTTCTT	−25.9	bol-miR55	GTTGGAGGAGGAGGAGGAGGA	−38.8
bol-miR28	GAGCGACTGTTTCTTCGTCGGA	−56.8			

### Expression profiling of known miRNAs

**Figure 2:**
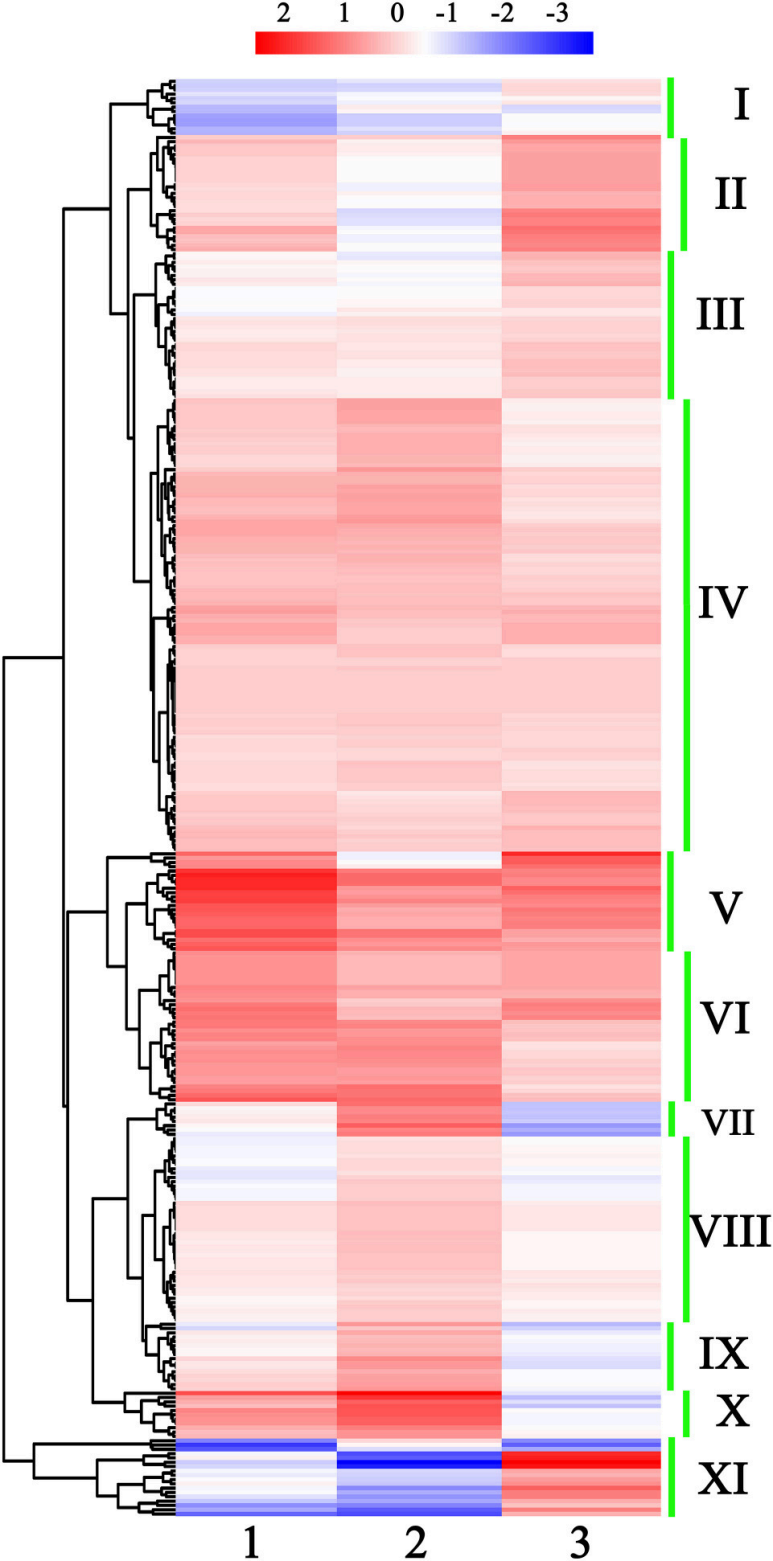
**Hierarchical clustering of all detected known miRNAs in early developmental stages of broccoli pollen. I to XI show the 11 miNRA groups clustered by their expression profiling**. The color scale at the top illustrated the relative expression levels of each miRNA in two arbitrary developmental phases of broccoli pollen from high (red) to low (blue). 1, 2, and 3 at the bottom of three columns represented the relative expression levels of each miRNA shown by Log_2_ (uninucleate microspores/trinucleate Pollen grains), Log_2_ (binucleate pollen grains/trinucleate pollen grains) and Log_2_ (uninucleate microspores/binucleate pollens) ratio, respectively.

miRNAs with similar expression patterns may have similar functions. Thus, the expression patterns of 333 conserved known miRNAs were further analyzed. According to the hierarchical clustering data, these 333 miRNAs could be roughly classified into 11 groups (Figure 2 and Table S7). miRNAs from the same family, such as the members of miR156/157, miR160, miR165, miR167, miR319, miR3955, and miR858, always belonged to the same group, implying that they exhibited similar expression patterns in the development of broccoli pollen.

### Differential expression of miRNAs in pollen development

**Figure 3:**
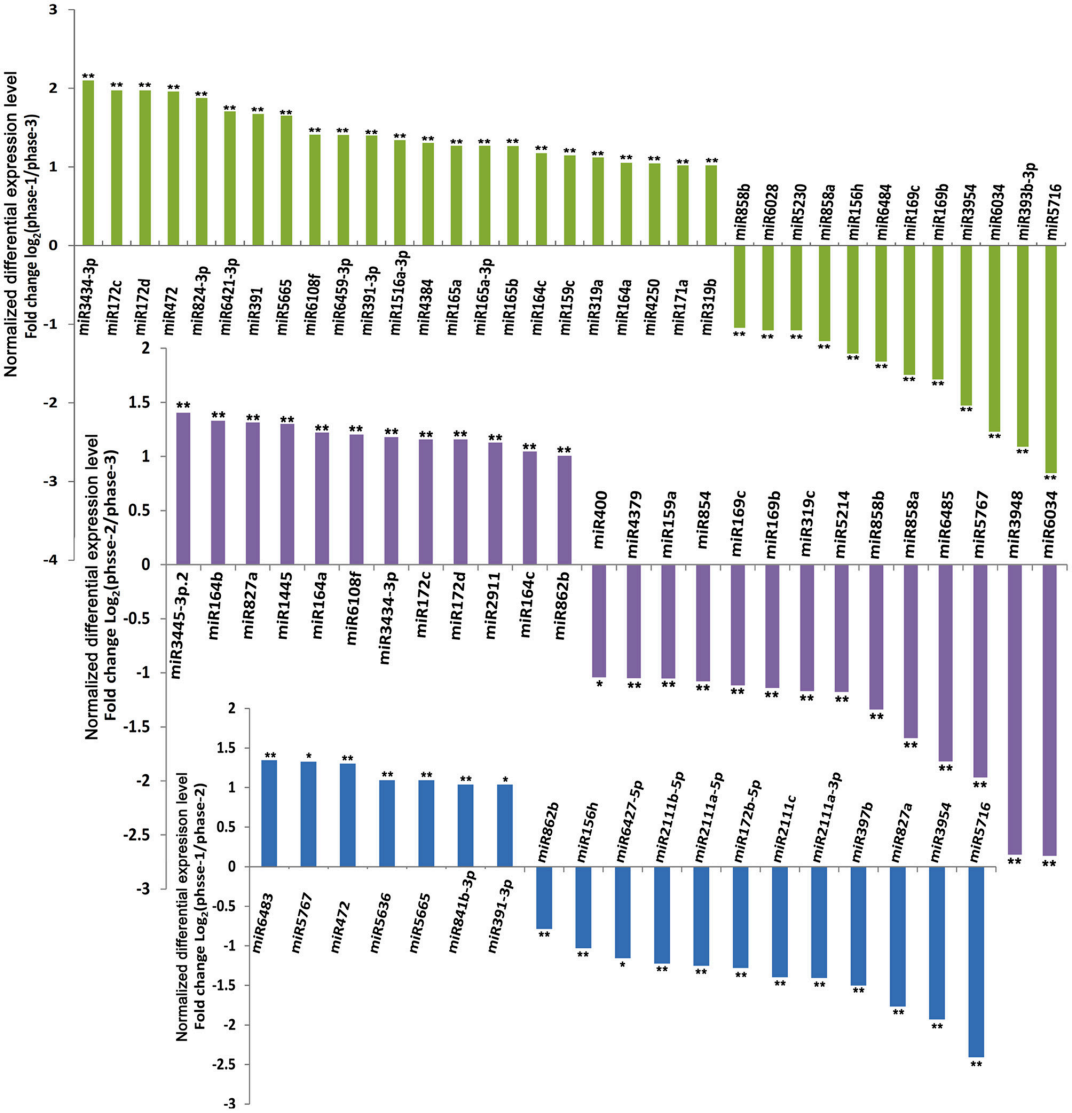
**Differential expression levels of known miRNAs in broccoli uninucleate microspores, binucleate pollen grains and trinucleate pollen grains**. ^**^ indicated the significantly differential expression level with corrected *P* < 0.01; ^*^ indicated the significantly differential expression level with corrected *P* < 0.05. phase-1, phase-2, and phase-3 indicated uninucleate microspores, binucleate pollen grains and trinucleate pollen grains, respectively.

**Figure 4:**
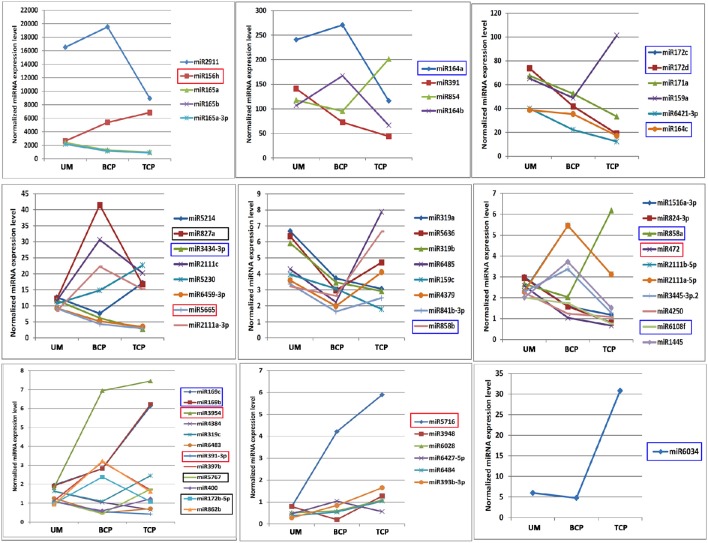
**Expression trends of known miRNAs which showed significantly differential expression levels in the three developmental phases of broccoli pollen**. miRNAs which showed significantly differential expression patterns in the uninucleate microspores, binucleate pollen grains and trinucleate pollens were marked by corresponding red, black and blue boxes, respectively. UM, BCP, and TCP indicated uninucleate microspores, binucleate pollen grains and trinucleate pollen grains, respectively. The significantly differentially expressed miRNAs are grouped by their expression levels.

Based on the expression levels and patterns of each miRNA, sixty of the 333 known conserved miRNAs were confirmed to exhibit significantly differential expression levels in the three different developmental phases of broccoli pollen (Figure 3 and Table S8). Pairwise comparison analysis indicated that 19 known miRNAs in the uninucleate microspores and binucleate pollen grains showed significantly differential expression levels. Among these known miRNAs, 7 miRNAs displayed higher expression levels, whereas the remaining other 12 known miRNAs displayed lower expression levels in the uninucleate microspores than those in the binucleate pollen grains. Compared with those in the trinucleate pollen grains, 35 known miRNAs with significantly differential expression levels were detected in the uninucleate microspores. The expression levels of miRNAs in the binucleate and trinucleate pollen grains were also compared. Twenty-six known miRNAs with significantly differential expression levels were detected in the two phases. Of these miRNAs, 12 had up-regulated expression in the binucleate phase and 14 showed down-regulated expression (Figure 3, Tables S8, S9). Further analysis indicated that the expression levels of miR156h, miR391, miR3954, miR472, miR5665, and miR5716 in the uninucleate microspores significantly differed from those in the binucleate and trinucleate pollen grains. MiR156 h and miR5716 showed considerably low expression levels in the uninucleate microspores, while four other miRNAs showed particularly high expression levels. In binucleate pollen grains, miR5767 showed particularly low expression levels, whereas miR172, miR827a, and miR862b showed specifically high expression levels. In the trinucleate pollen grains, the expression levels of 11 known miRNAs (miR164c, miR169b, miR169c, miR172c, miR172d, miR858a, miR164a, miR6034, miR6108f, miR858b, and miR3434), were significantly different from those in uninucleate microspores and binucleate pollen grains. MiR169b, miR169c, miR858a, miR6034, and miR858b showed considerably high expression levels in the trinucleate pollen grains, while the six other known miRNAs demonstrated evidently low expression levels (Figure 4, Table S8).

The expression trends of miRNAs with significantly differential expression levels were also analyzed. Nine of the 60 known miRNAs increased their expression levels with the development of pollen, while the expression levels of 22 miRNAs decreased. Approximately half of these miRNAs showed “V” or reversed “V” expression patterns. In addition, 22 of the 60 differentially expressed miRNAs belonged to seven miRNA families (miR164, miR165, miR169, miR172, miR2111, miR159/319, and miR858). Similarly, miRNAs from the same miRNA family exhibited similar expression trends (Figures 2, 4 and Table S7).

In 55 predicted novel miRNAs, the expression profiles of predicted novel miRNAs with read counts >20 were further observed. The results indicated that, except for seven novel miRNAs only detected in a specific developmental phase, four predicted novel miRNAs showed significantly differential expression levels in the three samples (Figure S5). Bol-miR01 showed similar expression levels in the uninucleate microspores and binucleate pollen grains but was not detected in the trinucleate pollen grains. Bol-miR03 was specifically and highly expressed in the binucleate pollen grains but was lowly expressed in both uninucleate microspores and trinucleate pollen grains. Bol-miR33 and bol-miR38 were undetected in the uninucleate microspores, while both were highly expressed in the binucleate and trinucleate pollen grains. The other four novel miRNAs all detected in three developmental phases did not exhibit significantly differential expression levels (Figure S5).

### Prediction of miRNA candidate targets

**Figure 5:**
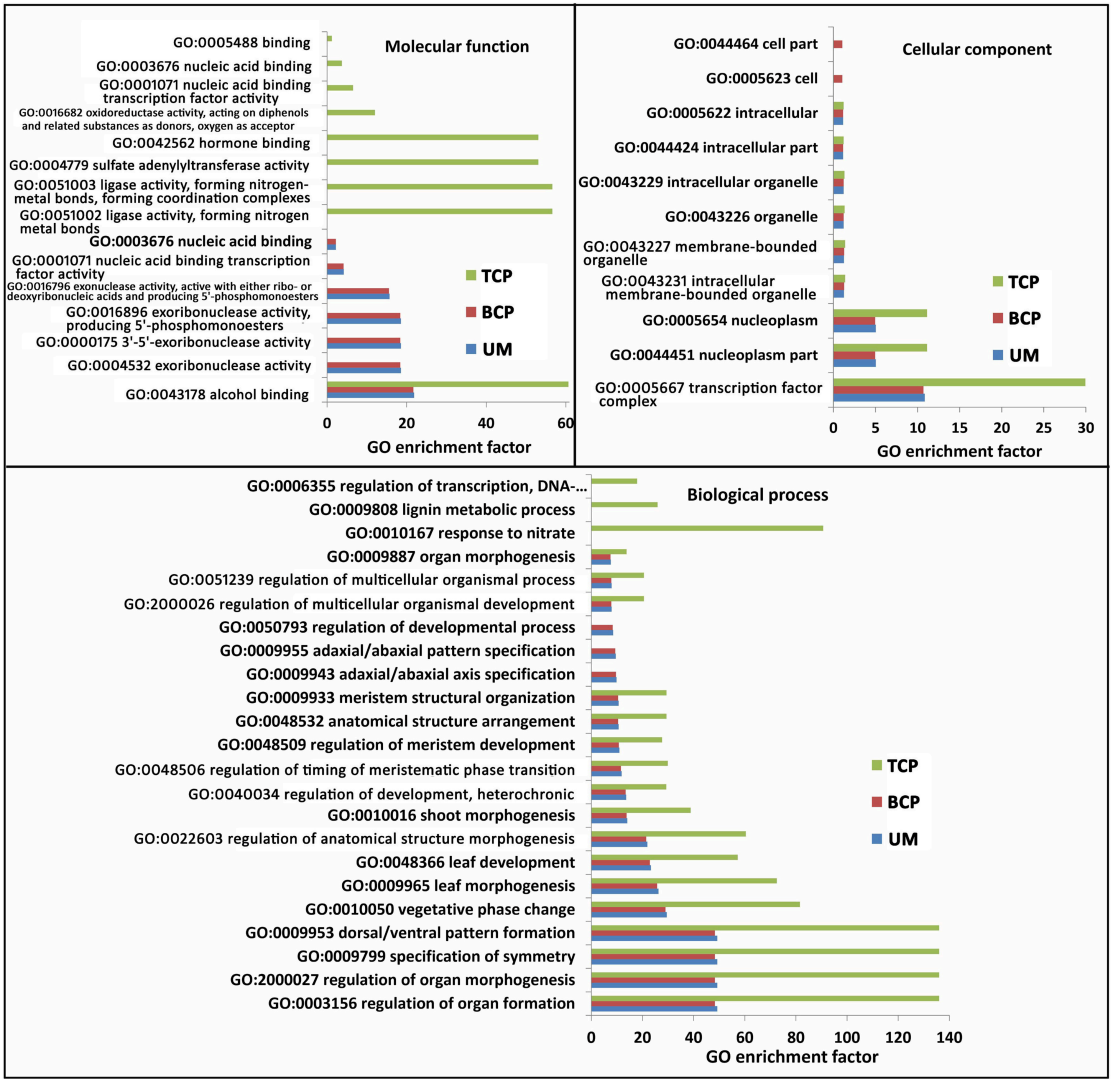
**Significantly enriched GO terms of known miRNA targets in the three developmental phases of broccoli pollen (corrected *P* < 0.05)**. Top 20 significantly enriched GO terms were showed in each developmental phases of broccoli pollen. UM, BCP, and TCP indicated the uninucleate microspores, binucleate pollen grains and trinucleate pollen grains, respectively.

A total of 552 target sites were identified for 127 conserved miRNAs. More than 10 putative genes were targeted by miR156, miR157, miR414, miR4993, miR5021, miR5137, miR5139, miR5215, miR5227, miR5631, miR5782, miR6443, and miR854, whereas only a few targets were detected for most miRNAs (Table S10). Homologous analysis and functional annotation of the putative targets were performed. The annotated targets were mainly associated with the regulation of plant growth and development (Table S10). All of the targets were subjected to GO analysis. The GO terms of the conserved miRNA targets from the uninucleate microspores and the two following developmental products of microspores were significantly enriched in 23 biological processes, 11 cellular components and 15 molecular functions. In biological processes, 17 GO terms, including GO:0003156 (regulation of organ formation) and GO:2000027 (regulation of organ morphogenesis), were enriched for the targets predicted in all three developmental phases. GO:0009943 (adaxial/abaxial axis specification), GO:0009955 (adaxial/abaxial pattern specification) and GO:0050793 (regulation of developmental process) were significantly enriched for the targets predicted in the uninucleate microspores and binucleate pollen grains. Three other GO terms were only significantly enriched for the targets predicted only in the trinucleate pollen grains. In cellular components, nine of the 11 GO terms were significantly enriched for the targets predicted in the three developmental phases. In molecular function, eight of the 15 GO terms were especially enriched for the targets predicted in the trinucleate pollen grains. Other GO terms from this section were specifically enriched for the targets predicted in the uninucleate microspores and binucleate pollen grains (Figure 5 and Table S11).

A total of 69 putative targets were predicted to be cleaved by 40 predicted novel miRNAs (Table S12). The annotated targets also underwent GO analysis. No significantly enriched GO terms were detected for the targets of novel miRNAs found in the uninucleate microspores. The GO terms associated with floral organ development, pollen development, postembryonic organ development and response to nitrate only significantly enriched for the targets predicted in the binucleate pollen grains. For the predicted miRNA targets in the trinucleate pollen grains, 10 significantly enriched GO terms mainly involved in biological processes were detected. The hormone binding GO term (GO:0042562) exhibited significant enrichment for the targets predicted in both the binucleate and trinucleate pollen grains (Figure S6).

### Validation of the expression patterns of miRNAs and miRNA targets

**Figure 6:**
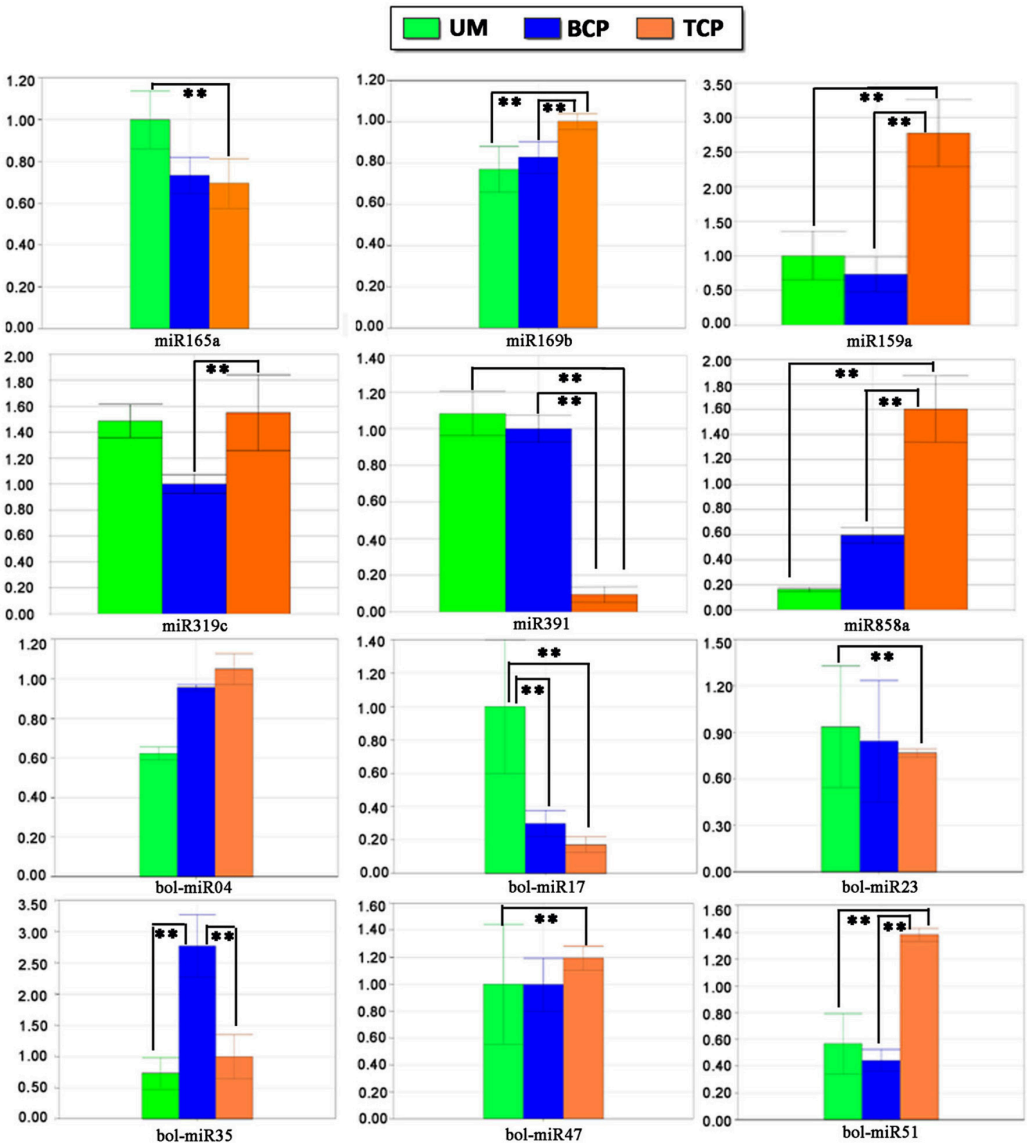
**Differential expression patterns of several known miRNAs and novel miRNAs in different developmental phases of broccoli pollen validated by quantitative stem-loop RT-PCR**. The small nuclear RNA U6 was used as an internal reference. ^**^ indicated the significantly differential expression level with *P* < 0.01. UM, BCP, and TCP indicated the uninucleate microspores, binucleate pollen grains, and trinucleate pollen gains, respectively.

Twelve conserved miRNAs and 25 of the 55 predicted novel miRNAs were randomly selected for stem-loop RT-PCR and re-sequencing validation. Most of these detected miRNAs could be amplified except for bol-miR10 and bol-miR21. Nevertheless, the precursor sequences of the two novel miRNAs were successfully amplified. Sequencing analysis confirmed that the mature miRNA and precursor sequences of bol-miR10 and bol-miR21 were consistent with those predicted by the bioinformatics method (Table 1). The expression patterns of six conserved miRNAs (miR165a, miR169b, miR159a, miR319c, miR391, and miR858a) and eight novel miRNAs (bol-miR04, bol-miR06, bol-miR17, bol-miR21, bol-miR23, bol-miR35, bol-miR47, and bol-miR51) were further explored by real-time quantitative stem-loop RT-PCR. The expression patterns of six conserved miRNAs and six of the eight novel miRNAs that were detected by qRT-PCR were highly consistent with the data produced by high-throughput sequencing (the coefficient of determination *R*^2^ > 0.86) (Figure 6 and Figure S7). In addition, the expression patterns of the 12 putative targets for bol-miR04, bol-miR17, bol-miR23, bol-miR34, and bol-miR51 were observed (Table S12). The expression trends of at least one putative target for each novel miRNA were confirmed to be negatively correlated with the expression levels of the corresponding miRNA (Figure S8).

### Validation of novel miRNA targets by modified 5′ RLM-RACE

**Figure 7:**
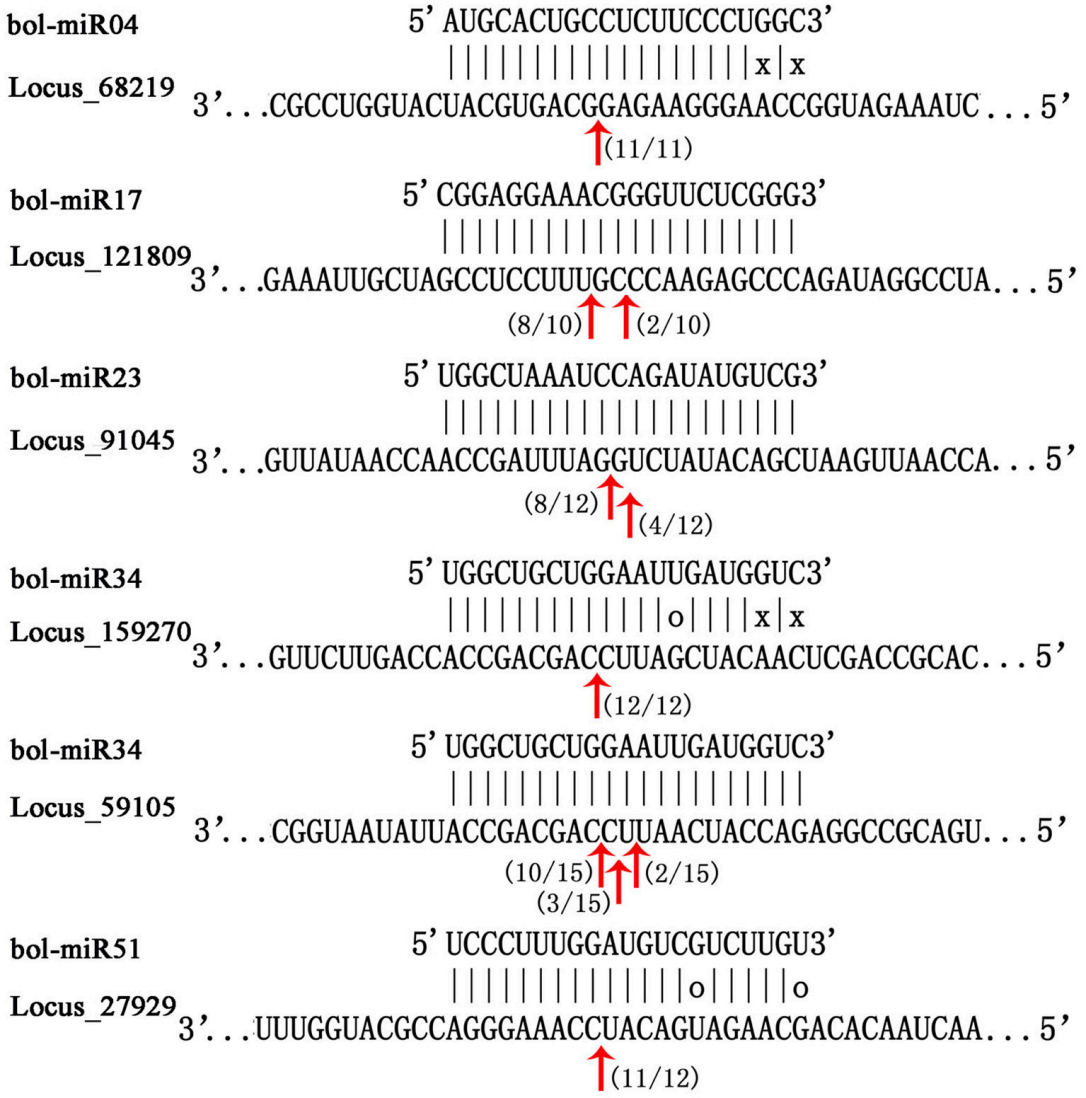
**Mapping of mRNA cleavage sites of novel miRNAs by modified 5′ RLM-RACE**. The miRNA sequence was at the top, and the target sequence was at the bottom. Perfectly complementary bases were shown connected by vertical dashes. G:U wobble pairing was indicated by circles, and other non-complementary bases were shown by “x.” The red arrows indicated the sites of cleavage, and numbers near the arrows indicated the fraction of cloned PCR products terminating at different positions. The target sequences could be found in Table S12.

The modified 5′ RLM-RACE method was conducted to observe the cleavage sites of 12 putative targets of several novel miRNAs. Consistent with the expression trend analysis, the targets, whose expression trend negatively correlated with their miRNAs, were successfully cleaved by the corresponding miRNAs mainly in the complementary region of the miRNA and mRNA sequences. The cleavage sites were predominant in the 10^th^ and 11^th^ nucleotides from the 5′ end of miRNAs (Figure 7).

## Discussion

Microspore development is an important step in generating fertile male gametophytes in flowering plants. In some crops, such as broccoli, maize, rapeseed and Chinese cabbage, isolated microspores can reprogram toward embryogenesis *in vitro* and generate homozygous double-haploid plants. This phenomenon has been widely utilized to obtain homozygous parental materials in crops (Ferrie and Caswell, 2011; Corral-Martínez and Seguí-Simarro, 2012; Yuan et al., 2015). Several investigations have explored the natural basis of microspore embryogenesis (Belmonte et al., 2010; Rodríguez-Serrano et al., 2012; Solís et al., 2012, 2015; Ahmadi et al., 2016). Microspores or early binucleate pollen grains were demonstrated to more easily undergo embryogenesis *in vitro* (Keller et al., 1987; Ferrie, 2003). However, the early events of microspore development, such as those occurring in the uninucleate microspores, binucleate pollen grains or trinucleate pollen grains, and the transition from one developmental phase to another, are poorly understood. Therefore, genome-wide small RNAs in three significantly different developmental phases of broccoli pollen were sequenced and further analyzed in this study.

An overview of the small RNA sequencing data indicated that the count and length distributions of these small RNAs were highly similar throughout the three developmental phases of broccoli pollen (Table S3 and Figure S2). However, pairwise comparisons revealed that only a small proportion of unique small RNAs were shared by two arbitrary developmental phases. Most unique small RNAs specifically expressed in a certain developmental phase (Figure S1). This finding indicated that the distributions of small RNA populations markedly varied in uninucleate microspores, binucleate pollen grains and trinucleate pollen grains. However, the roles of these small RNAs in this process remain largely unknown.

miRNAs have been extensively analyzed given their various functions in diverse biological processes (Carrington and Ambros, 2003; Chen, 2009). Unlike the distributions of whole small RNAs, most known miRNAs were detected in the three early developmental stages of broccoli pollen. A few miRNAs, such as the members of miR167, miR166, miR156/157, miR165, miR158, and miR168 families, all exhibited high expression abundance during microspore development (Figure S3). Similarly, most members of these miRNA families showed high expression levels in other developmental processes of other plant species (Chávez Montes et al., 2014; Baksa et al., 2015; Liu et al., 2015; Roy et al., 2016). These miRNAs are likely to highly evolutionarily conserved. By contrast, almost all newly predicted novel miRNAs showed low expression abundance during the development of broccoli pollen. Most novel miRNAs detected in other plants or in the diverse biological processes of different plants also showed low expression abundance. Furthermore, these novel miRNAs are usually specific to a species or developmental phase (Wei et al., 2011; Peng et al., 2012; Jiang et al., 2014; Li et al., 2015; Qu et al., 2016). These results indicated that the predicted novel miRNAs were evolutionarily young with important roles in maintaining species-specificity regardless of their expression levels. However, given the limitations of forward genetic research methods, only a few miRNAs have been observed over time since miRNA was first identified in *Caenorhabditis elegans* (Lee et al., 1993; Reinhart et al., 2000). miRNAs with low expression abundance are more difficultly detected. In recent years, an increasing number of miRNAs, including those with low expression levels, have been identified via second-generation sequencing technologies. These newly identified miRNAs provide crucial clues for further understanding the function of miRNAs, especially those of newly evolved miRNAs.

The differential expression of miRNAs was analyzed to further explore their possible roles in broccoli development. Sixty known miRNAs exhibited significantly differential expression levels during the development of broccoli pollen. Nineteen and 26 known miRNAs showed significantly different expression levels between the uninucleate microspores and binucleate pollen grains, and between the binucleate and trinucleate pollen grains, respectively. In addition, the expression trends of these differentially expressed miRNAs varied throughout pollen development. (Figures 3, 4 and Table S8). A series of miRNAs that are closely involved in the development of plant male gametophytes were identified in other plant species. Eighteen differentially expressed known miRNAs including miRNA159a, miR164a, miR172, miR319b, miR391a, and miR824-3p have been confirmed between the flower buds of the male sterile and male fertile lines of *Brassica campestris* ssp. *Chinensis*. Most of these miRNAs are implicated in pollen development (Jiang et al., 2014). Twenty-six conserved known miRNAs including miR159a, miR159c, miR169, miR171, miR172, miR319b, and miR391 showed significant differences in expression between mature pollen and inflorescence in Arabidopsis (Chambers and Shuai, 2009). In addition, 14 miRNAs exhibiting pollen-enriched expression, such as miR156, miR171a, and miR824, were also confirmed in Arabidopsis (Grant-Downton et al., 2009). Similarly, 202 known miRNAs and 75 novel miRNAs were confirmed to express in the developing pollen of rice, among which more than half of the miRNAs displayed pollen -or stage-specific expression (Wei et al., 2011). In maize, 40 conserved and 16 novel miRNAs displayed differential expression levels between mature and germinated pollen (Li et al., 2013).

Consistent with these previous investigations, the miRNAs including miR156, miRNA159a, miR159c, miR164a, miR169, miR171, miR172, miR319b, miR391a, and miR824-3p, which displayed significantly differential expression levels in the early stages of broccoli pollen development, also have been confirmed to involve in the development of male gametophytes in other plant species. It implied that these miRNAs should also function in the development of broccoli pollen. In addition, several known miRNAs, such as miR2111, miR2911, miR400, miR400, miR827, miR854, miR858, and miR862, have only been confirmed to show significantly differential expression in the present study. Moreover, miR159a, miR319c, miR2111, miR397, miR400, miR8483, miR6427, miR841, and miR854 showed only significantly differential expression levels between the uninucleate microspores and binucleate pollen grains, or the binucleate and trinucleate pollen grains. The known miRNAs and predicted novel miRNAs showing extremely specific expression patterns, for example showing extremely high expression level only in a certain developmental phase, were also identified. These findings provided more valuable clues to explore their functions in the early event of broccoli pollen development.

miRNAs, as important endogenous RNA regulators, cleave the target mRNAs or inhibit translation. Consequently, the identification and functional elucidation of miRNA targets are crucial to uncover the roles of miRNAs. In the present study, 552 targets for 127 conserved known miRNAs and 69 targets regulated by 40 predicted novel miRNAs were identified in broccoli. A few of them have been annotated as the homologs of corresponding known miRNA targets in other plants. The homologs of these target genes were also confirmed to be cleaved by corresponding miRNAs in broccoli. In addition, the quantitative expression analysis of 12 randomly selected novel miRNA targets indicated that the expression levels of over half of these targets (8/12) were negatively correlated with those of their corresponding miRNAs. These results were consistent with the fact that miRNAs mainly negatively regulate their targets (Carrington and Ambros, 2003). The cleavage sites of six of these targets were also identified by modified 5′ RLM-RACE. Consistent with previous investigations, the cleavage sites were predominant in the 10^th^ and 11^th^ nucleotide from the 5′ end of the miRNAs (Song et al., 2010; Li et al., 2015). These results demonstrated that identifying miRNA targets by bioinformatics analysis is feasible, although further experimental validations are still required. Further functional annotation and GO analysis of these miRNA targets indicated that the GO terms of all annotated targets mainly were significantly enriched in plant organ formation, morphogenesis and other early events of plant development (Figure 5 and Figure S6). The targets, especially those identified targets by 5′ RLM-RACE, provided significant clues to explore the function of miRNA-target module in broccoli pollen development.

In conclusion, the significantly differentially expressed known miRNAs and the novel miRNAs, combined with their potential targets, provide new insight into the early development of broccoli pollen.

## Author contributions

HL performed the experiments, analyzed the data and wrote the manuscript; YW performed the experiments; MW analyzed the data; LL performed the experiments; CJ performed the experiments; QZ performed the experiments; CC analyzed the data; WS analyzed the data and wrote the manuscript; CW designed the project, analyzed the data and wrote the manuscript.

### Conflict of interest statement

The authors declare that the research was conducted in the absence of any commercial or financial relationships that could be construed as a potential conflict of interest.
