# Supplementary material for: Small RNA Sequencing Reveals Differential miRNA Expression in the Early Development of Broccoli (Brassica oleracea var. italica) Pollen
Source: Front Plant Sci. 2017 Mar 24;8:404. doi: 10.3389/fpls.2017.00404 (PMC5364186; doi:10.3389/fpls.2017.00404)
Supplement: Supplementary file 1 [file Table1.DOCX]

**Small RNA sequencing reveals differential miRNA expression in the early development of broccoli (*Brassica oleracea* var. *italica*) pollen**

Hui Li^2^, Chuan Jin^1^, Yu Wang^1^, Mei Wu^1^, Lihong, Li^1^, Qingli Zhang^1^, Chengbin Chen^1^, Wenqin Song^1^, Chunguo Wang^1**^

^1^College of Life Sciences, Nankai University, Tianjin 300071, China;

^2^College of Horticulture and Landscape, Tianjin Agricultural University, Tianjin, 300384, China

**Corresponding author: email: [wangcg@nankai.edu.cn](mailto:wangcg@nankai.edu.cn); Telephone: 86-22-23508241; Fax: 86-22-23508800

Email address:

Hui Li：lihui@tjau.edu.cn; Yu Wang: 1581257798@qq.com; Mei Wu: alexmaymolecular@126.com; Lihong, Li: 348536673@qq.com; Chuan Jin: 15822076271@163.com; Qingli Zhang: 13553162779@163.com; Chengbin Chen: htg1979@163.com; Wenqin Song: songwenqin53@gmail.com

| Primers | Sequences (5’-3’) |
| --- | --- |
| RT and amplified primers used in known miRNA stem-loop RT-PCR | RT-miR156h: CTCAACTGGTGTCGTGGAGTCCGGCAATTCAGTTGAGGTGCTCTC |
|  | Forward-miR156h: ACACTCCAGCTGGGTTGACAGA |
|  | RT-miR159a: CTCAACTGGTGTCGTGGAGTCCGGCAATTCAGTTGAGTAGAGCTCC |
|  | Forward-miR159a: ACACTCCAGCTGGGTTTGGATT |
|  | RT-miR164c: CTCAACTGGTGTCGTGGAGTCCGGCAATTCAGTTGAGCGCACGTG |
|  | Forward-miR164c: ACACTCCAGCTGGGTGGAGAAG |
|  | RT-miR165a: CTCAACTGGTGTCGTGGAGTCCGGCAATTCAGTTGAGCGATCCAGA |
|  | Forward-miR165a: ACACTCCAGCTGGGAGGGGAAT |
|  | RT-miR169b:CTCAACTGGTGTCGTGGAGTCCGGCAATTCAGTTGAGGTAGCCGAA |
|  | Forward-miR169b: ACACTCCAGCTGGGTGGCAAGT |
|  | RT-miR172a: CTCAACTGGTGTCGTGGAGTCCGGCAATTCAGTTGAGTGTGAATCT |
|  | Forward-miR172a: ACACTCCAGCTGGGGTGGCATC |
|  | RT-miR319c: CTCAACTGGTGTCGTGGAGTCCGGCAATTCAGTTGAGGACTGGAC |
|  | Forward-miR319c: ACACTCCAGCTGGG GGAGATTC |
|  | RT-miR391: CTCAACTGGTGTCGTGGAGTCCGGCAATTCAGTTGAGGTAGGAGAG |
|  | Forward-miR391: ACACTCCAGCTGGGTGGTGACG |
|  | RT-miR397a: CTCAACTGGTGTCGTGGAGTCCGGCAATTCAGTTGAGCATCAACGC |
|  | Forward-miR397a: ACACTCCAGCTGGGTCATTGAG |
|  | RT-miR408: CTCAACTGGTGTCGTGGAGTCCGGCAATTCAGTTGAGGCCAGGGA |
|  | Forward-miR408: ACACTCCAGCTGGGATGCACTG |
|  | RT-miR858a: CTCAACTGGTGTCGTGGAGTCCGGCAATTCAGTTGAGAAGGTCGA |
|  | Forward-miR858a: ACACTCCAGCTGGGTTTCGTTGT |
|  | RT-miR168a: CTCAACTGGTGTCGTGGAGTCCGGCAATTCAGTTGAGTTCCCGAC |
|  | Forward-miR168a: ACACTCCAGCTGGGTCGCTTGGT |
|  | Universe reverse primer: CAACTGGTGTCGTGGAG |
|  | U6- forward: CTCGCTTCGGCAGCACA |
|  | U6- reverse: AACGCTTCACGAATTTGCGT |

**Supplementary Table S1** Primers used in present study.

| Primers | Sequences (5’-3’) |
| --- | --- |
| RT and amplified primers used in novel miRNA stem-loop RT-PCR | RT- Bro-miR01: CTCAACTGGTGTCGTGGAGTCCGGCAATTCAGTTGAGCTTCGAGCC |
|  | Forward- Bro-miR01: ACACTCCAGCTGGGGGAATGTTG |
|  | RT- Bro-miR02: CTCAACTGGTGTCGTGGAGTCCGGCAATTCAGTTGAGGATTGAAC |
|  | Forward- Bro-miR02: ACACTCCAGCTGGGAGATATTAG |
|  | RT- Bro-miR03: CTCAACTGGTGTCGTGGAGTCCGGCAATTCAGTTGAGTGCCATCC |
|  | Forward- Bro-miR03: ACACTCCAGCTGGGGGCCGTGGG |
|  | RT- Bro-miR04: CTCAACTGGTGTCGTGGAGTCCGGCAATTCAGTTGAGGCCAGGGA |
|  | Forward- Bro-miR04: ACACTCCAGCTGGGATGCACTGC |
|  | RT- Bro-miR06: CTCAACTGGTGTCGTGGAGTCCGGCAATTCAGTTGAGAATCCTCGCC |
|  | Forward- Bro-miR06: ACACTCCAGCTGGGAATGAAATAA |
|  | RT- Bro-miR07: CTCAACTGGTGTCGTGGAGTCCGGCAATTCAGTTGAGGGCTCCGA |
|  | Forward- Bro-miR07: ACACTCCAGCTGGGTGCATCAAC |
|  | RT- Bro-miR09: CTCAACTGGTGTCGTGGAGTCCGGCAATTCAGTTGAGCTGCAATG |
|  | Reverse –Bro-miR09: ACACTCCAGCTGGGTCTGAAACT |
|  | RT- Bro-miR10: CTCAACTGGTGTCGTGGAGTCCGGCAATTCAGTTGAG CACGACAA |
|  | Forward- Bro-miR10: ACACTCCAGCTGGG TAGCTAGTA |
|  | RT- Bro-miR11: CTCAACTGGTGTCGTGGAGTCCGGCAATTCAGTTGAGAAGGAAAA |
|  | Forward- Bro-miR11: ACACTCCAGCTGGGCTTCGTGTA |
|  | RT- Bro-miR15: CTCAACTGGTGTCGTGGAGTCCGGCAATTCAGTTGAG GTTGATCT |
|  | Reverse –Bro-miR15: ACACTCCAGCTGGGACCCTTCTC |
|  | RT- Bro-miR17: CTCAACTGGTGTCGTGGAGTCCGGCAATTCAGTTGAGCCCGAGAAC |
|  | Forward- Bro-miR17: ACACTCCAGCTGGGCGGAGGAAA |
|  | RT- Bro-miR20: CTCAACTGGTGTCGTGGAGTCCGGCAATTCAGTTGAGCCACCATC |
|  | Forward- Bro-miR20: ACACTCCAGCTGGGGAGGTATGG |
|  | RT- Bro-miR21: CTCAACTGGTGTCGTGGAGTCCGGCAATTCAGTTGAGCGCCGGGA |
|  | Forward- Bro-miR21: ACACTCCAGCTGGGGCTTGTCTC |
|  | RT- Bro-miR23: CTCAACTGGTGTCGTGGAGTCCGGCAATTCAGTTGAGCGACATATC |
|  | Forward- Bro-miR23: ACACTCCAGCTGGGTGGCTAAAT |
|  | RT- Bro-miR25: CTCAACTGGTGTCGTGGAGTCCGGCAATTCAGTTGAGGATAAAAAC |
|  | Forward- Bro-miR25: ACACTCCAGCTGGGGCCATGGCGGAAGAGTTTTTATC |
|  | RT- Bro-miR30: CTCAACTGGTGTCGTGGAGTCCGGCAATTCAGTTGAG GGGTTACT |
|  | Forward- Bro-miR30: ACACTCCAGCTGGGGGACGTGCT |
|  | RT- Bro-miR33: CTCAACTGGTGTCGTGGAGTCCGGCAATTCAGTTGAGTCACAGATG |
|  | Forward- Bro-miR33: ACACTCCAGCTGGGACAGCTCTG |
|  | RT- Bro-miR38: CTCAACTGGTGTCGTGGAGTCCGGCAATTCAGTTGAGGTTGGCCAA |
|  | Reverse –Bro-miR38: ACACTCCAGCTGGGCCAGATCTG |
|  | RT- Bro-miR40: CTCAACTGGTGTCGTGGAGTCCGGCAATTCAGTTGAGTCCTTCCA |
|  | Reverse –Bro-miR40: ACACTCCAGCTGGGCAGCTGTAG |
|  | RT- Bro-miR42: CTCAACTGGTGTCGTGGAGTCCGGCAATTCAGTTGAGCTTGATGC |
|  | Forward- Bro-miR42: ACACTCCAGCTGGGGGAGAGTCG |
|  | RT- Bro-miR47: CTCAACTGGTGTCGTGGAGTCCGGCAATTCAGTTGAGTGCCAAAACT |
|  | Forward- Bro-miR47: ACACTCCAGCTGGGCAAGTTGTAG |
|  | RT- Bro-miR48: CTCAACTGGTGTCGTGGAGTCCGGCAATTCAGTTGAGTAGCCTTACA |
|  | Forward- Bro-miR48: ACACTCCAGCTGGGAGGAGACTGT |
|  | RT- Bro-miR50: CTCAACTGGTGTCGTGGAGTCCGGCAATTCAGTTGAGTTGATCTG |
|  | Reverse –Bro-miR50: ACACTCCAGCTGGGCACCCTTCT |
|  | RT- Bro-miR51: CTCAACTGGTGTCGTGGAGTCCGGCAATTCAGTTGAGACAAGACGA |
|  | Reverse –Bro-miR51: ACACTCCAGCTGGGTCCCTTTGG |
|  | RT- Bro-miR52: CTCAACTGGTGTCGTGGAGTCCGGCAATTCAGTTGAGCCCGATGG |
|  | Reverse –Bro-miR52: ACACTCCAGCTGGGCGTCATTTG |
|  | Universe reverse primer: CAACTGGTGTCGTGGAG |
|  | U6- forward: CTCGCTTCGGCAGCACA |
|  | U6- reverse: AACGCTTCACGAATTTGCGT |
| Primers used to amplify miRNA precursors | Forward- Broc-miR10 (P): AGTCTTATCATATGGCACCATTTG |
|  | Reverse –Broc-miR10 (P): GATCTCGAGACACGACAACATCTAC |
|  | Forward- Broc-miR21 (P): GACATGTTCGTGCTGTGGTCT |
|  | Reverse –Broc-miR21 (P): TACAAGAAATCGCCGGGAT |

| Primers | Target ID | Sequences (5’-3’) |
| --- | --- | --- |
| Primers used to amplify miRNA targets in qRT-PCR | Locus_67914 | Forward Broc-miR04-target1: CAACGAAGGGAAGAGGCAGTG |
|  |  | Reverse Broc-miR04-target1: AGTGAAGCAAGAGCGGTGGTG |
|  | Locus_68219^a^ | Forward Broc-miR04-target1: AGGGAAGAGGCAGTGCATCAT |
|  |  | Reverse Broc-miR04-target1: TGTAGGCCAACCCACAGCGTT |
|  | Locus_30305 | Forward Broc-miR17-target1: TCGTATTCTGATGAATCGGGTCG |
|  |  | Reverse Broc-miR17-target1: AGGAAACGGGTTCTCGGGTCT |
|  | Locus_62553 | Forward Broc-miR17-target2: CCGTTTCCTCCGATCGTTAAA |
|  |  | Reverse Broc-miR17-target2: TCTCCCCTATCCAGTCCACAA |
|  | Locus_88455 | Forward Broc-miR17-target3: CCGCATAGAAGACTCGCTGGAA |
|  |  | Reverse Broc-miR17-target3: TAACTGCTCGTCGCTGATACAC |
|  | Locus_121809^a^ | Forward Broc-miR17-target4: GCTTGACCTCGTATTCGGATGA |
|  |  | Reverse Broc-miR17-target4: AAACGGGTTCTCGGGTCTATCC |
|  | Locus_91045^a^ | Forward Broc-miR23-target: ATTTCTTGAATGGTTCCACCCC |
|  |  | Reverse Broc-miR23-target : TATTTTGGTTTCAATCCTTGGT |
|  | Locus_59105^a^ | Forward Broc-miR34-target1: GGTCCACGAATGTCCAATGAT |
|  |  | Reverse Broc-miR34-target1: GAGAAAGACCAAGTCCTCCAC |
|  | Locus_78115 | Forward Broc-miR34-target2: GGTTTCACCTTCCCACAGTTC |
|  |  | Reverse Broc-miR34-target2: AACCCATGTAGAAAGCAGACG |
|  | Locus_159270^a^ | Forward Broc-miR34-target3: GATTCCAGCAGCCACCAGTTC |
|  |  | Reverse Broc-miR34-target3: TACTACTCCCTCGTGATCCTTTC |
|  | Locus_27929^a^ | Forward Broc-miR51-target1: TCGGTCCTGCGAGTGTCCTGTA |
|  |  | Reverse Broc-miR51-target1:GAGAACGCGGCCAAACTAGAAA |
|  | Locus_54855 | Forward Broc-miR51-target2: TACACCTAATTTCGGAAAGGAC |
|  |  | Reverse Broc-miR51-target2: GAATGGATATGAGGGAAGACAG |
|  |  | Forward Actin: GCTCCTCTTAACCCAAAGGC |
|  |  | Reverse Actin: CACACCATCACCAGAATCCAGC |

Notes: Sequences of the detected targets could be found in Table S11; “a” indicated the cleavage sites of these targets were identified by modified 5’RLM-RACE.

| Primers | Sequences (5’-3’) |
| --- | --- |
| Primers used in 5’RACE | Broc-miR04-target2 GSP1: TCGGATTATAGTTAAACACTAAAAC |
|  | Broc-miR04-target2 GSP2: GTTAAACACTAAAACGTCACCAG |
|  | Broc-miR017-target3 GSP1: ATTTAACTCTTAAATCTCAAAAAGAT |
|  | Broc-miR17-target3 GSP2: CTCTTAAATCTCAAAAAGATTATTT |
|  | Broc-miR23-target GSP1: ATATTTTCTCTCCTTTTCATGTGT |
|  | Broc-miR23-target GSP2: CTCCTTTTCATGTGTTTATCAAC |
|  | Broc-miR34-target1 GSP1: ATCGGAGAAGAGTTCATGGCTG |
|  | Broc-miR34-target1 GSP2: AAGAGTTCATGGCTGGAGTTTTGTT |
|  | Broc-miR34-target3 GSP1: TCCAGGCAAGGGAGGGAACAAT |
|  | Broc-miR34-target3 GSP2: CAAGGGAGGGAACAATAATCTTCAC |
|  | Broc-miR51-target GSP1: GTTGTCAGGCTGTGAGAGTTTAAAC |
|  | Broc-miR51-target GSP2: GCTGTGAGAGTTTAAACAAGCTTG |
|  | 5’ RACE Outer Primer: CATGGCTACATGCTGACAGCCTA |
|  | 5’ RACE Inner Primer: CGCGGATCCACAGCCTACTGATGATCAGTCGATG |

| Primers | Sequences (5’-3’) |
| --- | --- |
| Primers used in small RNA sequencing | RNA 5’ Adapter: GUUCAGAGUUCUACAGUCCGACGAUC |
|  | RNA 3’ Adapter: TGGAATTCTCGGGTGCCAAGG |
|  | RNA RT Primer: GCCTTGGCACCCGAGAATTCCA |
|  | RNA PCR Primer (RP1):  AATGATACGGCGACCACCGAGATCTACACGTTCAGAGTTCTACAGTCCGA |
|  | RNA PCR Primer, Index 2:  CAAGCAGAAGACGGCATACGAGATACATCGGTGACTGGAGTTCCTTGGCACCCGAGAATTCCA |
|  | RNA PCR Primer, Index 3:  CAAGCAGAAGACGGCATACGAGATGCCTAAGTGACTGGAGTTCCTTGGCACCCGAGAATTCCA |
|  | RNA PCR Primer, Index 5:  CAAGCAGAAGACGGCATACGAGATCACTGTGTGACTGGAGTTCCTTGGCACCCGAGAATTCCA |
|  | RNA PCR Primer, Index 6:  CAAGCAGAAGACGGCATACGAGATATTGGCGTGACTGGAGTTCCTTGGCACCCGAGAATTCCA |
|  | RNA PCR Primer, Index 7:  CAAGCAGAAGACGGCATACGAGATGATCTGGTGACTGGAGTTCCTTGGCACCCGAGAATTCCA |
|  | RNA PCR Primer, Index 8:  CAAGCAGAAGACGGCATACGAGATTCAAGTGTGACTGGAGTTCCTTGGCACCCGAGAATTCCA |
