# Supplementary material for: Small RNA Sequencing Reveals Differential miRNA Expression in the Early Development of Broccoli (Brassica oleracea var. italica) Pollen
Source: Front Plant Sci. 2017 Mar 24;8:404. doi: 10.3389/fpls.2017.00404 (PMC5364186; doi:10.3389/fpls.2017.00404)
Supplement: Supplementary file 2 [file Table2.docx]

**Small RNA sequencing reveals differential miRNA expression in the early development of broccoli (*Brassica oleracea* var. *italica*) pollen**

Hui Li^2^, Chuan Jin^1^, Yu Wang^1^, Mei Wu^1^, Lihong, Li^1^, Qingli Zhang^1^, Chengbin Chen^1^, Wenqin Song^1^, Chunguo Wang^1**^

^1^College of Life Sciences, Nankai University, Tianjin 300071, China;

^2^College of Horticulture and Landscape, Tianjin Agricultural University, Tianjin, 300384, China

**Corresponding author: email: [wangcg@nankai.edu.cn](mailto:wangcg@nankai.edu.cn); Telephone: 86-22-23508241; Fax: 86-22-23508800

Email address:

Hui Li：lihui@tjau.edu.cn; Yu Wang: 1581257798@qq.com; Mei Wu: alexmaymolecular@126.com; Lihong, Li: 348536673@qq.com; Chuan Jin: 15822076271@163.com; Qingli Zhang: 13553162779@163.com; Chengbin Chen: htg1979@163.com; Wenqin Song: songwenqin53@gmail.com

**Supplementary Table S2** Purity of uninucleate microspores, binucleate pollen grains and trinucleate pollen grains isolated from the flower buds with different sizes in broccoli.

| Microscope visual fields | Number  of all cells | Number of microspores | Microscope visual fields | Number of all cells | Number of  binucleate pollen grains | Microscope visual fields | Number of all cells | Number of  trinucleate pollen grains |
| --- | --- | --- | --- | --- | --- | --- | --- | --- |
| Field-01M | 32 | 32 | Field-01B | 45 | 43 | Field-01T | 70 | 65 |
| Field-02M | 46 | 45 | Field-02B | 56 | 53 | Field-02T | 81 | 77 |
| Field-03M | 38 | 38 | Field-03B | 40 | 37 | Field-03T | 76 | 75 |
| Field-04M | 59 | 57 | Field-04B | 63 | 60 | Field-04T | 75 | 74 |
| Field-05M | 30 | 28 | Field-05B | 57 | 56 | Field-05T | 69 | 67 |
| Field-06M | 55 | 54 | Field-06B | 49 | 49 | Field-06T | 84 | 81 |
| Field-07M | 67 | 67 | Field-07B | 68 | 62 | Field-07T | 66 | 63 |
| Field-08M | 60 | 59 | Field-08B | 65 | 63 | Field-08T | 73 | 69 |
| Field-09M | 71 | 69 | Field-09B | 51 | 48 | Field-09T | 59 | 56 |
| Field-10M | 48 | 48 | Field-10B | 72 | 68 | Field-10T | 78 | 75 |
|  |  |  | Field-01B | 70 | 69 | Field-01T | 84 | 82 |
|  |  |  | Field-12B | 76 | 70 | Field-12T | 62 | 62 |
| Totals | 506 | 497 | Totals | 712 | 678 | Totals | 877 | 846 |
| Purity of microspores | 98.2% | | Purity of binucleate pollen grains | 95.2% | | Purity of trinucleate pollen grains | 96.5% | |
