# Supplementary material for: Small RNA Sequencing Reveals Differential miRNA Expression in the Early Development of Broccoli (Brassica oleracea var. italica) Pollen
Source: Front Plant Sci. 2017 Mar 24;8:404. doi: 10.3389/fpls.2017.00404 (PMC5364186; doi:10.3389/fpls.2017.00404)
Supplement: Supplementary file 3 [file Table3.DOCX]

**Small RNA sequencing reveals differential miRNA expression in the early development of broccoli (*Brassica oleracea* var. *italica*) pollen**

Hui Li^2^, Chuan Jin^1^, Yu Wang^1^, Mei Wu^1^, Lihong, Li^1^, Qingli Zhang^1^, Chengbin Chen^1^, Wenqin Song^1^, Chunguo Wang^1**^

^1^College of Life Sciences, Nankai University, Tianjin 300071, China;

^2^College of Horticulture and Landscape, Tianjin Agricultural University, Tianjin, 300384, China

**Corresponding author: email: [wangcg@nankai.edu.cn](mailto:wangcg@nankai.edu.cn); Telephone: 86-22-23508241; Fax: 86-22-23508800

Email address:

Hui Li：lihui@tjau.edu.cn; Yu Wang: 1581257798@qq.com; Mei Wu: alexmaymolecular@126.com; Lihong, Li: 348536673@qq.com; Chuan Jin: 15822076271@163.com; Qingli Zhang: 13553162779@163.com; Chengbin Chen: htg1979@163.com; Wenqin Song: songwenqin53@gmail.com

**Supplementary Table S3** Overview of Solexa sequencing data in early developmental stages of broccoli pollen.

|  | Uninucleate microspores | | Binucleate pollen grains | | Trinucleate pollen grains | |
| --- | --- | --- | --- | --- | --- | --- |
|  | Unique reads | Total reads | Unique reads | Total reads | Unique reads | Total reads |
| Raw reads |  | 21659809 |  | 20439333 |  | 21429804 |
| High quality reads |  | 21599330 |  | 20381795 |  | 21370131 |
| Clean reads (18-30 nt) | 7160154 (100%) | 21429050 (100%) | 6299777 (100%) | 20173912 (100%) | 6952258 (100%) | 21209081 (100%) |
| Exon antisense | 8698 (0.12%) | 17897 (0.08%) | 7912 (0.13%) | 18284 (0.09%) | 8298 (0.12%) | 20403 (0.1%) |
| Exon sense | 16949 (0.24%) | 23105 (0.11%) | 16640 (0.26%) | 22457 (0.11%) | 14967 (0.22%) | 21153 (0.1%) |
| Intron antisense | 1128 (0.02%) | 1848  (0.01%) | 1070 (0.02%) | 1704 (0.01%) | 1243 (0.02%) | 1999 (0.01%) |
| Intron sense | 1665 (0.02%) | 2254  (0.01%) | 1449 (0.02%) | 1944 (0.01%) | 1673 (0.02%) | 2148 (0.01%) |
| miRNA | 21689 (0.30%) | 1697489 (7.92%) | 20783 (0.32%) | 2074148 (10.28%) | 20608 (0.30%) | 2409462 (11.36%) |
| rRNA | 126786 (1.77%) | 2630823 (12.28%) | 120770 (1.92%) | 2499192 (12.39%) | 113376 (1.63%) | 2262582 (10.67%) |
| Repeat | 4337 (0.06%) | 18926 (0.09%) | 4225 (0.07%) | 21006 (0.1%) | 4646 (0.07%) | 17213 (0.08%) |
| snRNA | 4849 (0.07%) | 17436 (0.08%) | 4672 (0.07%) | 15834 (0.08%) | 4528 (0.07%) | 12845 (0.06%) |
| snoRNA | 2508 (0.04%) | 10059 (0.05%) | 2300 (0.04%) | 8198 (0.04%) | 2002 (0.03%) | 5679 (0.03%) |
| tRNA | 16208 (0.23%) | 2116805 (9.88%) | 16716 (0.27%) | 2437973 (12.08%) | 14084 (0.2%) | 2167573 (10.22%) |
| Unannotated reads | 6955337 (97.13%) | 14892408 (69.5%) | 6103240 (96.88%) | 13073172 (64.8%) | 6766833 (97.33%) | 14288024  (67.37%) |
