# Supplementary material for: Small RNA Sequencing Reveals Differential miRNA Expression in the Early Development of Broccoli (Brassica oleracea var. italica) Pollen
Source: Front Plant Sci. 2017 Mar 24;8:404. doi: 10.3389/fpls.2017.00404 (PMC5364186; doi:10.3389/fpls.2017.00404)
Supplement: Supplementary file 6 [file Table6.DOCX]

**Small RNA sequencing reveals differential miRNA expression in the early development of broccoli (*Brassica oleracea* var. *italica*) pollen**

Hui Li^2^, Chuan Jin^1^, Yu Wang^1^, Mei Wu^1^, Lihong, Li^1^, Qingli Zhang^1^, Chengbin Chen^1^, Wenqin Song^1^, Chunguo Wang^1**^

^1^College of Life Sciences, Nankai University, Tianjin 300071, China;

^2^College of Horticulture and Landscape, Tianjin Agricultural University, Tianjin, 300384, China

**Corresponding author: email: [wangcg@nankai.edu.cn](mailto:wangcg@nankai.edu.cn); Telephone: 86-22-23508241; Fax: 86-22-23508800

Email address:

Hui Li：lihui@tjau.edu.cn; Yu Wang: 1581257798@qq.com; Mei Wu: alexmaymolecular@126.com; Lihong, Li: 348536673@qq.com; Chuan Jin: 15822076271@163.com; Qingli Zhang: 13553162779@163.com; Chengbin Chen: htg1979@163.com; Wenqin Song: songwenqin53@gmail.com

**Supplementary Table S6** The expression patterns of predicted novel miRNAs in early developmental stages of broccoli pollen.

| Novel  miRNAs | UM | BCP | TCP | Novel  miRNAs | UM | BCP | TCP |
| --- | --- | --- | --- | --- | --- | --- | --- |
| bol-miR01 | ▲ | ▲ | ▬ | bol-miR29 | ▬ | ▲ | ▬ |
| bol-miR02 | ▲ | ▲ | ▲ | bol-miR30 | ▬ | ▲ | ▲ |
| bol-miR03 | ▲ | ▲ | ▬ | bol-miR31 | ▬ | ▲ | ▬ |
| bol-miR04 | ▲ | ▲ | ▲ | bol-miR32 | ▬ | ▲ | ▬ |
| bol-miR05 | ▲ | ▬ | ▬ | bol-miR33 | ▬ | ▲ | ▲ |
| bol-miR06 | ▲ | ▬ | ▬ | bol-miR34 | ▬ | ▲ | ▬ |
| bol-miR07 | ▲ | ▲ | ▲ | bol-miR35 | ▬ | ▲ | ▬ |
| bol-miR08 | ▲ | ▬ | ▬ | bol-miR36 | ▬ | ▲ | ▬ |
| bol-miR09 | ▲ | ▲ | ▲ | bol-miR37 | ▬ | ▲ | ▬ |
| bol-miR10 | ▲ | ▬ | ▬ | bol-miR38 | ▬ | ▲ | ▲ |
| bol-miR11 | ▲ | ▬ | ▬ | bol-miR39 | ▬ | ▲ | ▬ |
| bol-miR12 | ▲ | ▬ | ▬ | bol-miR40 | ▬ | ▲ | ▬ |
| bol-miR13 | ▲ | ▬ | ▬ | bol-miR41 | ▬ | ▲ | ▬ |
| bol-miR14 | ▲ | ▬ | ▬ | bol-miR42 | ▬ | ▲ | ▬ |
| bol-miR15 | ▲ | ▬ | ▬ | bol-miR43 | ▬ | ▬ | ▲ |
| bol-miR16 | ▲ | ▬ | ▬ | bol-miR44 | ▬ | ▬ | ▲ |
| bol-miR17 | ▲ | ▬ | ▬ | bol-miR45 | ▬ | ▬ | ▲ |
| bol-miR18 | ▲ | ▲ | ▲ | bol-miR46 | ▬ | ▬ | ▲ |
| bol-miR19 | ▲ | ▬ | ▬ | bol-miR47 | ▬ | ▬ | ▲ |
| bol-miR20 | ▲ | ▬ | ▬ | bol-miR48 | ▬ | ▬ | ▲ |
| bol-miR21 | ▲ | ▬ | ▬ | bol-miR49 | ▬ | ▬ | ▲ |
| bol-miR22 | ▲ | ▲ | ▲ | bol-miR50 | ▬ | ▬ | ▲ |
| bol-miR23 | ▲ | ▬ | ▬ | bol-miR51 | ▬ | ▬ | ▲ |
| bol-miR24 | ▲ | ▲ | ▬ | bol-miR52 | ▬ | ▬ | ▲ |
| bol-miR25 | ▲ | ▲ | ▬ | bol-miR53 | ▬ | ▬ | ▲ |
| bol-miR26 | ▬ | ▲ | ▬ | bol-miR54 | ▬ | ▬ | ▲ |
| bol-miR27 | ▬ | ▲ | ▲ | bol-miR55 | ▬ | ▬ | ▲ |
| bol-miR28 | ▬ | ▲ | ▬ |  |  |  |  |

Notes: Triangle indicated that the miRNAs were detected in corresponding developmental phases of broccoli pollen. Short line indicated that the miRNAs were not detected in the corresponding developmental phases. The different colors of triangle and short line showed the different expression patterns of miRNAs. UM, BCP and TCP indicated uninucleate microspores, binucleate pollen grains and trinucleate pollen grains, respectively.
