# Supplementary material for: Small RNA Sequencing Reveals Differential miRNA Expression in the Early Development of Broccoli (Brassica oleracea var. italica) Pollen
Source: Front Plant Sci. 2017 Mar 24;8:404. doi: 10.3389/fpls.2017.00404 (PMC5364186; doi:10.3389/fpls.2017.00404)
Supplement: Supplementary file 8 [file Table8.docx]

**Small RNA sequencing reveals differential miRNA expression in the early development of broccoli (*Brassica oleracea* var. *italica*) pollen**

Hui Li^2^, Chuan Jin^1^, Yu Wang^1^, Mei Wu^1^, Lihong, Li^1^, Qingli Zhang^1^, Chengbin Chen^1^, Wenqin Song^1^, Chunguo Wang^1**^

^1^College of Life Sciences, Nankai University, Tianjin 300071, China;

^2^College of Horticulture and Landscape, Tianjin Agricultural University, Tianjin, 300384, China

**Corresponding author: email: [wangcg@nankai.edu.cn](mailto:wangcg@nankai.edu.cn); Telephone: 86-22-23508241; Fax: 86-22-23508800

Email address:

Hui Li：lihui@tjau.edu.cn; Yu Wang: 1581257798@qq.com; Mei Wu: alexmaymolecular@126.com; Lihong, Li: 348536673@qq.com; Chuan Jin: 15822076271@163.com; Qingli Zhang: 13553162779@163.com; Chengbin Chen: htg1979@163.com; Wenqin Song: songwenqin53@gmail.com

**Supplementary Table S8** Expression characteristic of differentially expressed known miRNAs detected in early developmental stages of broccoli pollen.

| Known miRNAs | Normalized expression levels | | | Significance level | | |
| --- | --- | --- | --- | --- | --- | --- |
|  | UM | BCP | TCP | UM vs TCP | UM vs BCP | BCP vs TCP |
| miR156h | 2634.6945 | 5380.9098 | 6833.6294 | ** | ** |  |
| miR3954 | 1.82 | 6.9397 | 7.4496 | ** | ** |  |
| miR472 | 2.5666 | 1.0409 | 0.6601 | ** | ** |  |
| miR5665 | 9.1931 | 4.3125 | 2.9233 | ** | ** |  |
| miR5716 | 0.7933 | 4.2134 | 5.8937 | ** | ** |  |
| miR391-3p | 1.12 | 0.5453 | 0.4243 | ** | ** |  |
| miR165b | 2150.912 | 1145.7867 | 894.2867 | ** |  |  |
| miR159c | 3.9666 | 3.0733 | 1.7917 | ** |  |  |
| miR319a | 6.6732 | 3.7177 | 3.0647 | ** |  |  |
| miR319b | 5.9265 | 3.4698 | 2.9233 | ** |  |  |
| miR165a | 2366.5538 | 1277.3427 | 981.1363 | ** |  |  |
| miR171a | 67.4785 | 52.3944 | 33.2405 | ** |  |  |
| miR391 | 140.9302 | 72.5194 | 44.1792 | ** |  |  |
| miR4250 | 2.2399 | 1.2392 | 1.0844 | ** |  |  |
| miR4384 | 1.6333 | 1.0409 | 0.6601 | ** |  |  |
| miR5230 | 10.7331 | 14.8211 | 22.679 | ** |  |  |
| miR6028 | 0.5133 | 0.5948 | 1.0844 | ** |  |  |
| miR6484 | 0.3733 | 0.5453 | 1.0373 | ** |  |  |
| miR1516a-3p | 2.9866 | 1.5862 | 1.1787 | ** |  |  |
| miR165a-3p | 2172.7981 | 1160.707 | 902.255 | ** |  |  |
| miR393b-3p | 0.28 | 0.8427 | 1.6502 | ** |  |  |
| miR6421-3p | 39.9924 | 22.1573 | 12.2589 | ** |  |  |
| miR6459-3p | 9.2398 | 5.2047 | 3.4891 | ** |  |  |
| miR824-3p | 2.9399 | 1.5862 | 0.8015 | ** |  |  |
| miR164c | 38.6858 | 35.3427 | 17.1153 | ** |  | ** |
| miR169b | 1.9133 | 2.8254 | 6.2237 | ** |  | ** |
| miR169c | 1.96 | 2.8254 | 6.1294 | ** |  | ** |
| miR172c | 73.6384 | 41.8858 | 18.7655 | ** |  | ** |
| miR172d | 73.6384 | 41.8362 | 18.7655 | ** |  | ** |
| miR858a | 2.6599 | 2.0323 | 6.1766 | ** |  | ** |
| miR164a | 240.7013 | 270.6466 | 116.1295 | ** |  | ** |
| miR6034 | 5.9732 | 4.7586 | 30.7887 | ** |  | ** |
| miR6108f | 2.0066 | 1.7349 | 0.7544 | ** |  | ** |
| miR858b | 3.2199 | 2.6272 | 6.6481 | ** |  | ** |
| miR3434-3p | 11.7131 | 6.1961 | 2.7347 | ** |  | ** |
| miR397b | 1.12 | 3.1724 | 1.6974 |  | ** |  |
| miR6483 | 1.26 | 0.4957 | 0.7072 |  | ** |  |
| miR2111c | 11.6197 | 30.6336 | 20.2272 |  | ** |  |
| miR5636 | 6.3465 | 2.9741 | 4.715 |  | ** |  |
| miR2111a-5p | 2.2866 | 5.4526 | 3.1119 |  | ** |  |
| miR2111b-5p | 2.3333 | 5.4526 | 3.1119 |  | ** |  |
| miR6427-5p | 0.4667 | 1.0409 | 0.5658 |  | ** |  |
| miR2111a-3p | 8.3998 | 22.2565 | 15.1822 |  | ** |  |
| miR841b-3p | 3.3599 | 1.6358 | 2.4989 |  | ** |  |
| miR5767 | 1.12 | 0.4461 | 1.7445 |  | ** | ** |
| miR827a | 12.1797 | 41.4892 | 16.691 |  | ** | ** |
| miR862b | 0.9333 | 3.222 | 1.6031 |  | ** | ** |
| miR172b-5p | 0.98 | 2.3793 | 1.0844 |  | ** | ** |
| miR159a | 65.2385 | 48.8254 | 101.2774 |  |  | ** |
| miR164b | 106.8643 | 167.3944 | 66.5753 |  |  | ** |
| miR319c | 1.6333 | 1.0905 | 2.4518 |  |  | ** |
| miR1445 | 2.0066 | 3.7177 | 1.5088 |  |  | ** |
| miR2911 | 16510.9979 | 19520.0118 | 8931.5044 |  |  | ** |
| miR3948 | 0.7933 | 0.1983 | 1.273 |  |  | ** |
| miR400 | 1.0733 | 0.5948 | 1.2259 |  |  | ** |
| miR4379 | 3.5933 | 1.9828 | 4.102 |  |  | ** |
| miR5214 | 12.6464 | 7.5841 | 17.1625 |  |  | ** |
| miR6485 | 4.2932 | 2.2306 | 7.874 |  |  | ** |
| miR854 | 117.8774 | 95.222 | 201.2346 |  |  | ** |
| miR3445-3p | 2.2866 | 3.3707 | 1.273 |  |  | ** |

Notes: ** indicated the significantly differential expression level with corrected *P-*value < 0.01. UM, BCP and TCP indicated the uninucleate microspores, binucleate and trinucleate pollen grains, respectively.
