# Supplementary material for: Small RNA Sequencing Reveals Differential miRNA Expression in the Early Development of Broccoli (Brassica oleracea var. italica) Pollen
Source: Front Plant Sci. 2017 Mar 24;8:404. doi: 10.3389/fpls.2017.00404 (PMC5364186; doi:10.3389/fpls.2017.00404)
Supplement: Supplementary file 13 [file Presentation1.pdf]

Small RNA sequencing reveals differential miRNA expression in the early development of broccoli (*Brassica oleracea* var. *italica*) pollen

Hui Li[2], Chuan Jin[1], Yu Wang[1], Mei Wu[1], Lihong, Li[1], Qingli Zhang[1], Chengbin Chen[1], Wenqin Song[1], Chunguo Wang[1**]

[1]College of Life Sciences, Nankai University, Tianjin 300071, China;

[2]College of Horticulture and Landscape, Tianjin Agricultural University, Tianjin, 300384, China

**Corresponding author: email: wangcg@nankai.edu.cn; Telephone: 86-22-23508241; Fax: 86-22-23508800

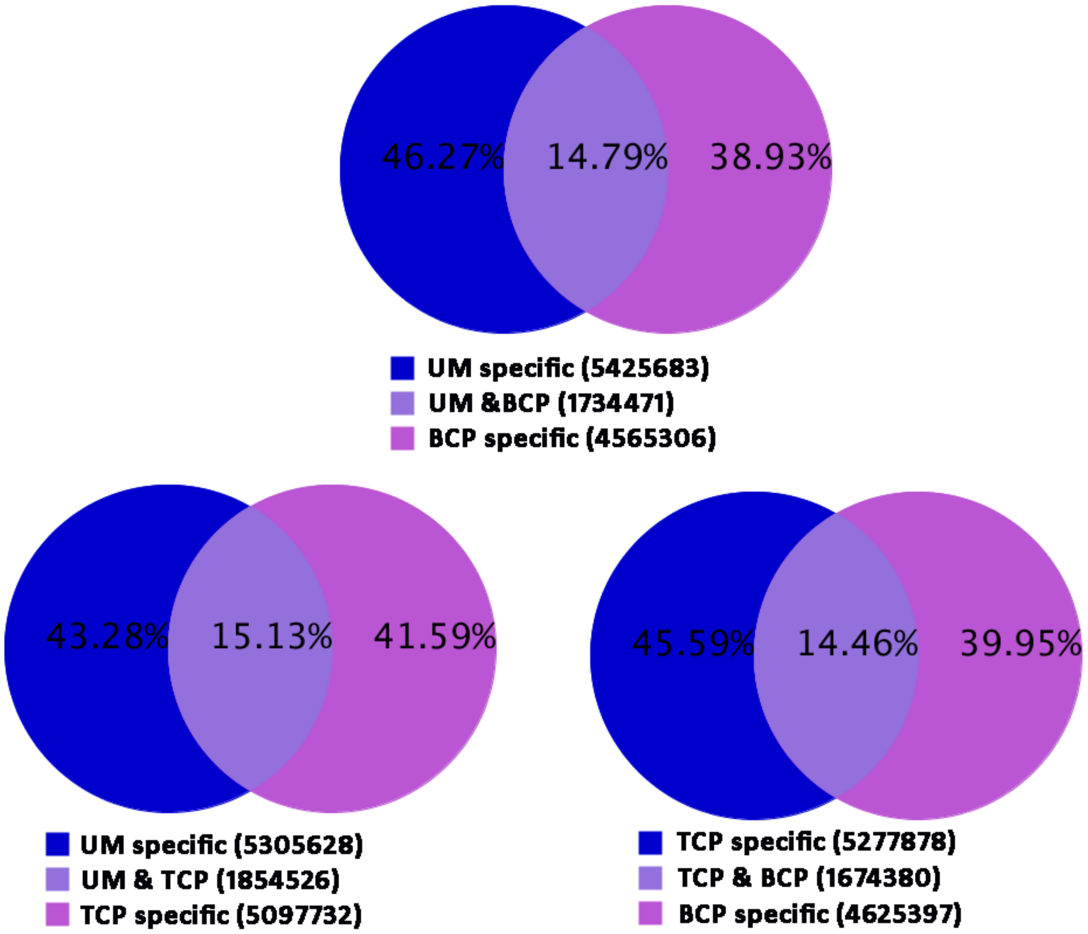

Supplementary Figure S1 Venn diagrams of unique small RNA distribution by pairwise comparison in early developmental stages of broccoli pollen. UM, BCP and TCP indicated the uninucleate microspores, binucleate pollen grains and trinucleate pollen grains, respectively. The number in the brackets indicated the count of unique small RNAs.

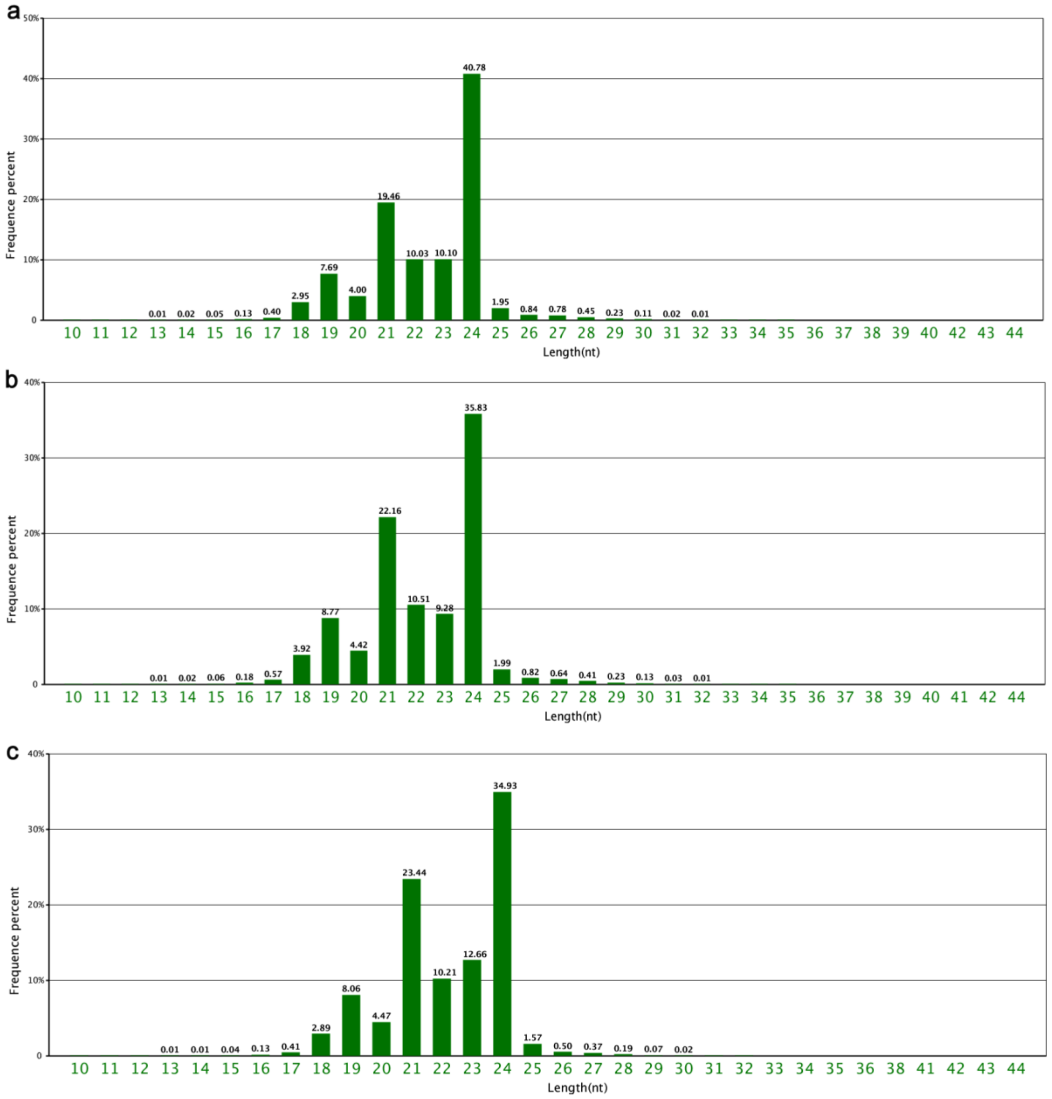

Supplementary Figure S2 Length distribution of small RNAs in early developmental stages of broccoli pollen. a, b and c indicated the broccoli uninucleate microspores, binucleate pollen grains and trinucleate pollen grains, respectively.

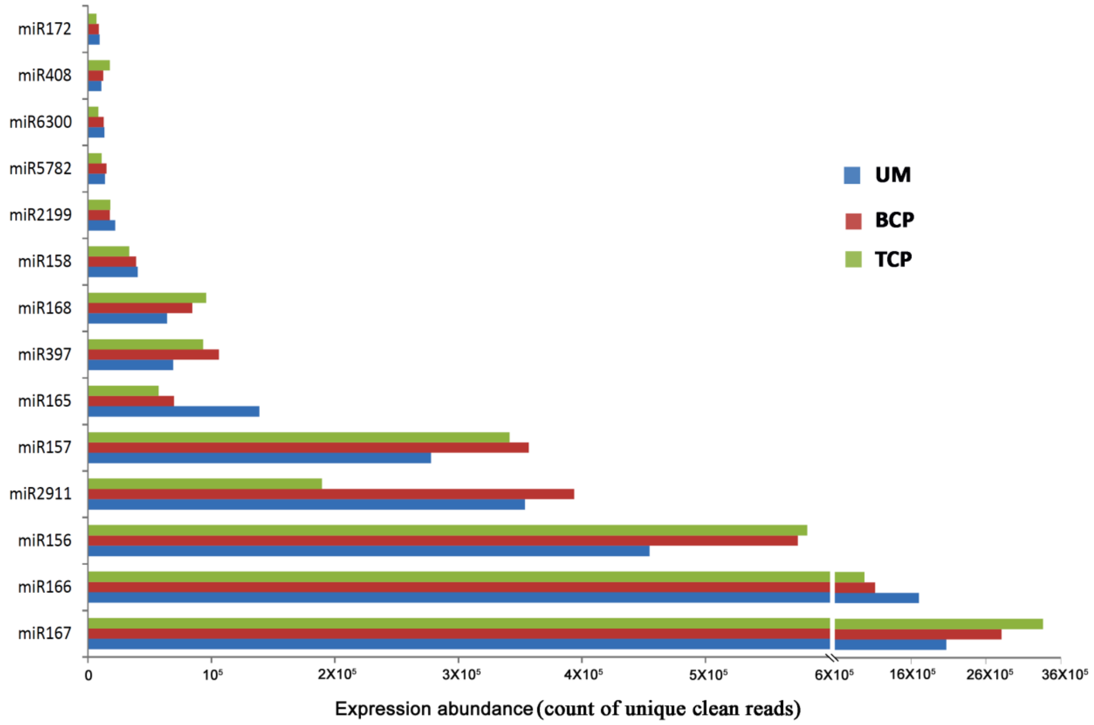

Supplementary Figure S3 Expression abundance patterns of representative known miRNA families in early developmental stages of broccoli pollen. The expression abundance data of other detected known miRNA families were showed in Table S5. UM, BCP and TCP indicated the uninucleate microspores, binucleate pollen grains and trinucleate pollen grains, respectively.

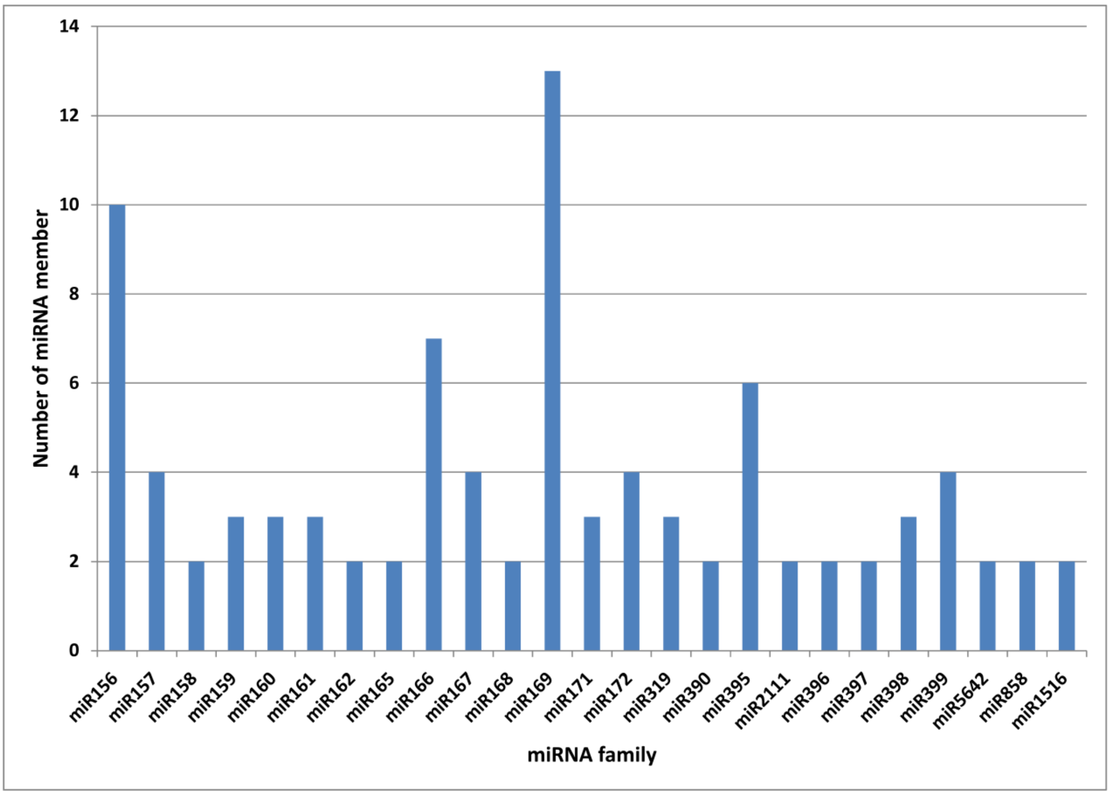

Supplementary Figure S4. The detected members of known miRNA families in the three developmental phases of broccoli microspores. Only those miRNA families containing ⩾ 2 members were showed.

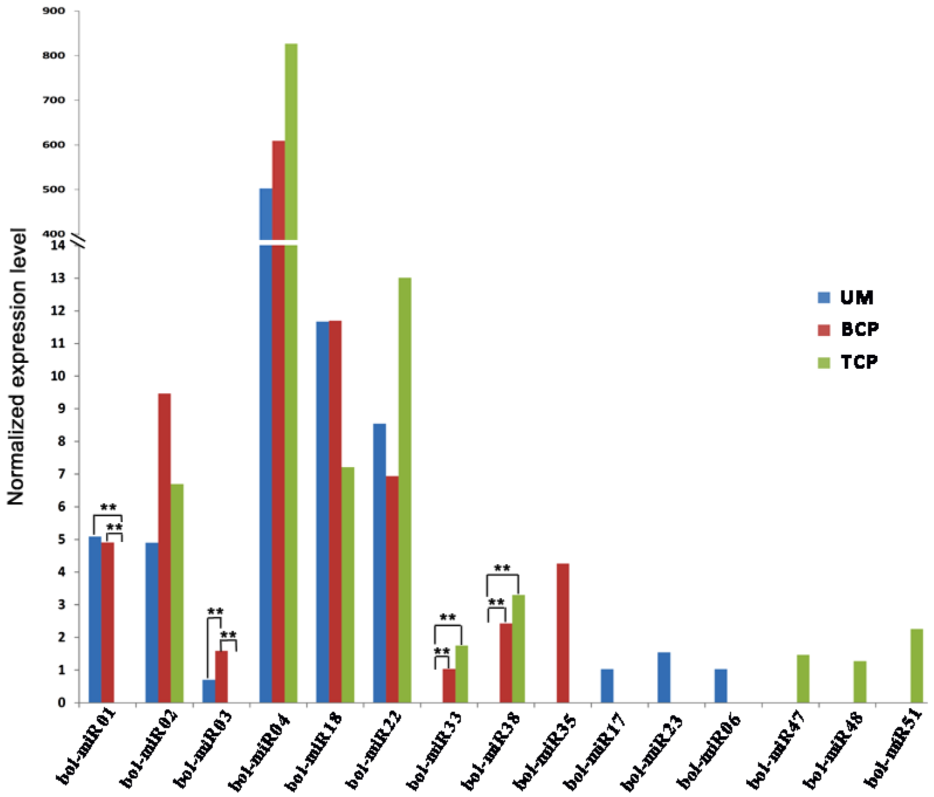

Supplementary Figure S5 Differential expression patterns of several predicted novel miRNAs (read count >20) in the different developmental phases of broccoli pollen. ** indicated the significantly differential expression level with corrected *P*-value < 0.01. UM, BCP and TCP indicated the uninucleate microspores, binucleate pollen grains and trinucleate pollen grains.

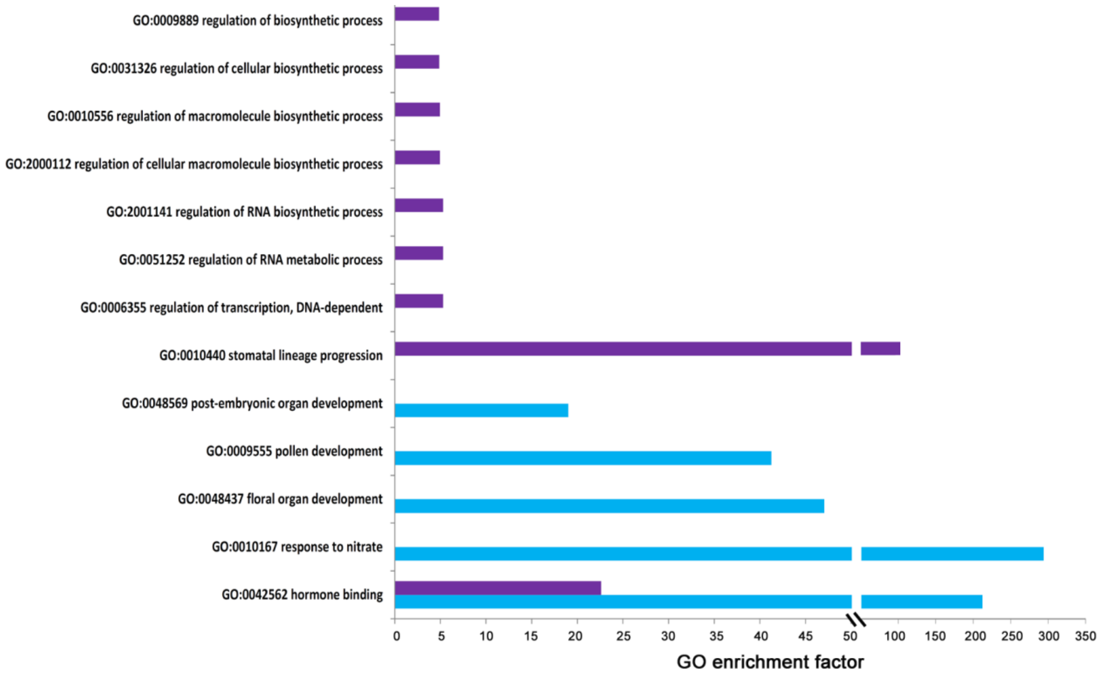

Supplementary Figure S6 GO analysis of predicted novel miRNA targets in broccoli uninucleate microspores, binucleate pollen grains and trinucleate pollen grains. Blue and purple bars indicated the significantly enriched GO terms for the targets predicted in the binucleate pollen grains and trinucleate pollen grains, respectively. The significant level was assessed by corrected *P*-value ($P<0.05$).

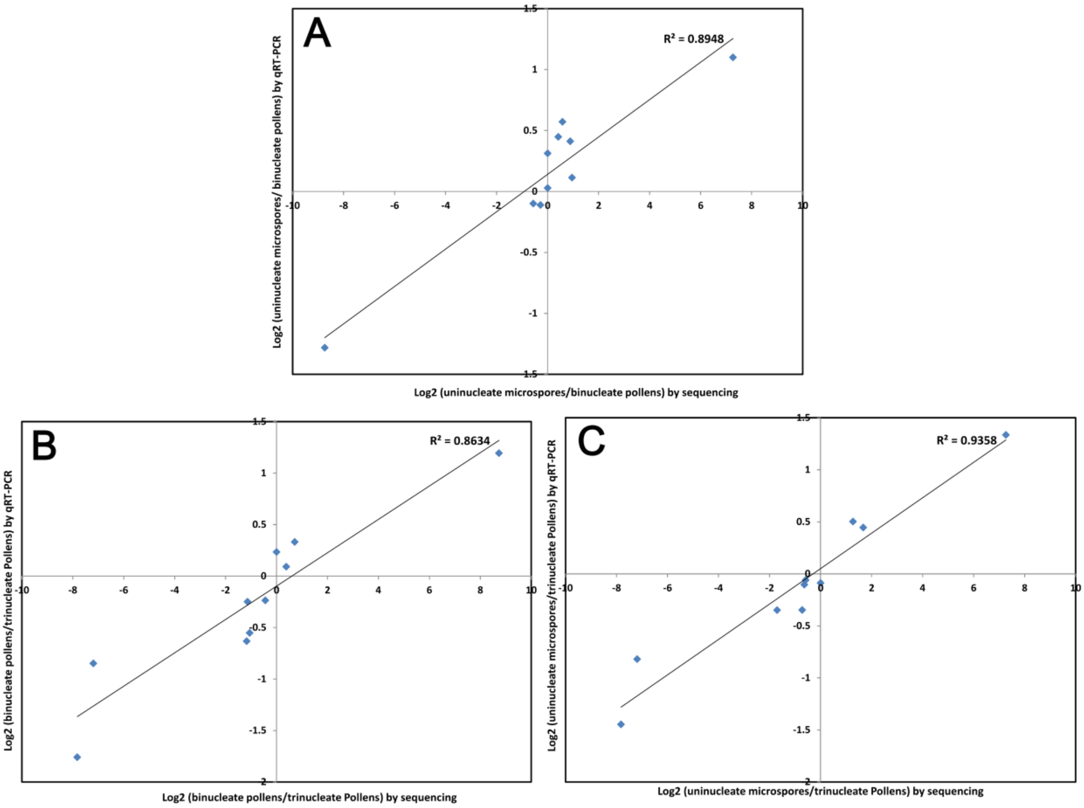

Supplementary Figure S7 Correlation of miRNA expression levels detected by small RNA sequencing and qRT-PCR analysis. A, B and C indicated the correlation of normalized relative expression levels detected by small RNA sequencing and qRT-PCR in three sets of pairwise comparison, which were uninucleate microspores vs. binucleate pollens (A), binucleate pollens vs. trinucleate pollens (B) and uninucleate microspores vs. trinucleate pollens (C), respectively. Y axis represented the relative expression levels detected by qRT-PCR. X axis represented the relative expression levels detected by small RNA sequencing.

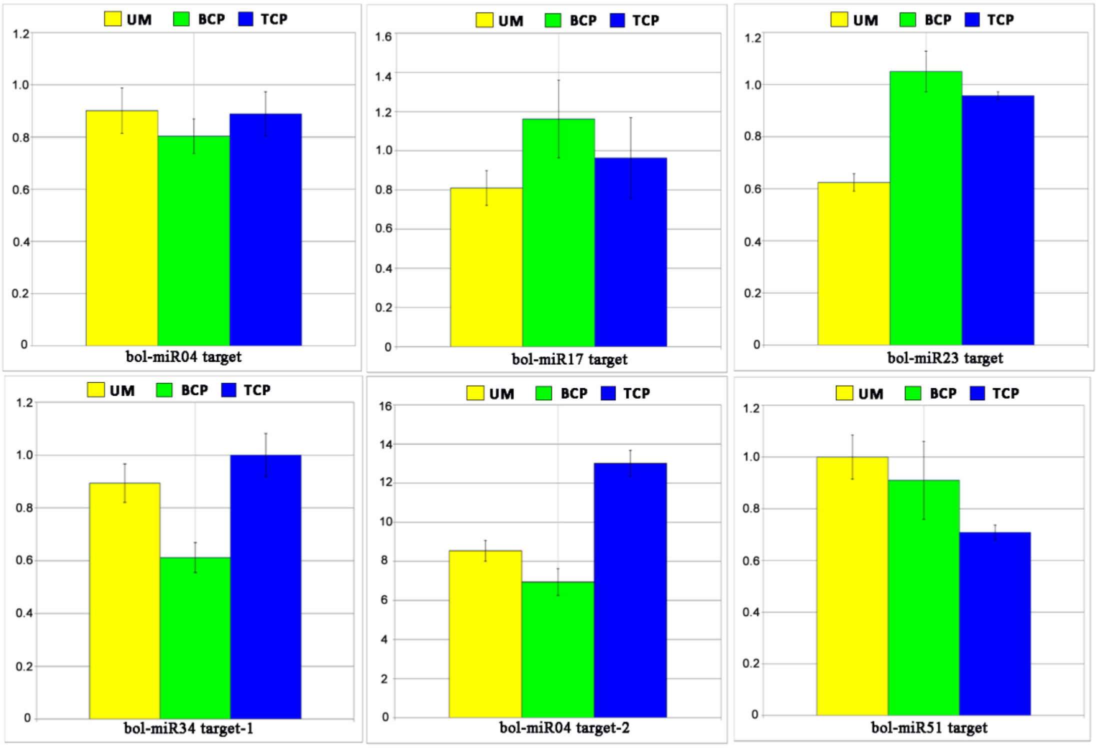

Supplementary Figure S8 Differential expression patterns of several novel miRNA targets in early developmental stages of broccoli pollen identified by qRT-PCR. The target sequences and annotated information showed in Table S12. UM, BCP and TCP indicated the uninucleate microspores, binucleate pollen grains and trinucleate pollen grains, respectively.
